# Distinct microbiological signatures associated with triple negative breast cancer

**DOI:** 10.1038/srep15162

**Published:** 2015-10-15

**Authors:** Sagarika Banerjee, Zhi Wei, Fei Tan, Kristen N. Peck, Natalie Shih, Michael Feldman, Timothy R. Rebbeck, James C. Alwine, Erle S. Robertson

**Affiliations:** 1 Department of Microbiology, University of Pennsylvania, 201 E Johnson Pavilion, 3610 Hamilton Walk, Philadelphia, PA 19104, USA; 2 Department of Computer Science, College of Computing Sciences, New Jersey Institute of Technology, GITC 4400, University Heights, Newark, NJ 07102, USA; 3 Department of Pathology and Laboratory Medicine, University of Pennsylvania, 3400 Spruce Street, Philadelphia, PA 19104, USA; 4 Department of Biostatistics and Epidemiology, University of Pennsylvania, 217 Blockley Hall, 423 Guardian Drive, Philadelphia, PA 19104, USA; 5 Department of Cancer Biology, University of Pennsylvania, 314 Biomedical Research Building, 421 Curie Blvd. Philadelphia, PA 19104, USA

## Abstract

Infectious agents are the third highest human cancer risk factor and may have a greater role in the origin and/or progression of cancers, and related pathogenesis. Thus, knowing the specific viruses and microbial agents associated with a cancer type may provide insights into cause, diagnosis and treatment. We utilized a pan-pathogen array technology to identify the microbial signatures associated with triple negative breast cancer (TNBC). This technology detects low copy number and fragmented genomes extracted from formalin-fixed paraffin embedded archival tissues. The results, validated by PCR and sequencing, define a microbial signature present in TNBC tissue which was underrepresented in normal tissue. Hierarchical clustering analysis displayed two broad microbial signatures, one prevalent in bacteria and parasites and one prevalent in viruses. These signatures demonstrate a new paradigm in our understanding of the link between microorganisms and cancer, as causative or commensal in the tumor microenvironment and provide new diagnostic potential.

The estimated number of new cancer cases in the United States for 2015 is about 1.6 million, with over 500,000 deaths (http://www.cancer.org)^1^. Infection with one or more viruses or microorganisms is the third highest contributor to the development of cancer^2^,^3^ accounting for at least 20% of tumors^3^. Ten viruses (Papillomavirus, Hepatitis B or C, Polyomaviruses like BK, JC and Merkel Cell Polyomavirus, Epstein-Barr Virus, Human Herpesvirus 8/Kaposi Sarcoma associated Herpesvirus, and T-cell Leukemia Virus type 1 and type-2), one bacterium (*Helicobacter pylori*), and two helminthes (*Schistosomes* and liver flukes) have been found to be major contributors to human cancers as etiological agents^3^. Given the many viruses and other microorganisms that are hosted by humans it is likely that their association with cancer is underestimated due to heretofore unrecognized infections or mechanisms^3^. For example, persistent infection by one or more infectious agents, resulting in inflammation or alteration of cellular processes, may be involved in the carcinogenic process^4^. Alternatively, the tumor microenvironment may provide a specialized niche in which these organisms can persist in a way that is difficult in normal tissue. In either case the identification of unique microbial signatures associated with specific cancers is essential for our understanding of the interplay between the microbiome and cancer, and for diagnosis.

Breast cancer is one of the most prevalent cancers: in 2015 an estimated 200,000 new cases will be diagnosed in the US resulting in over 40,000 deaths (http://www.cancer.org)^1^. Breast cancers are categorized on the basis of presence or absence of certain hormone and growth receptors. There are 4 major types: Endocrine receptor (estrogen or progesterone receptor) positive, human epidermal growth factor receptor 2 (HER2) positive, triple positive (estrogen, progesterone and HER2 receptor positive) and triple negative (absence of estrogen, progesterone and HER2 receptors) (http://www.cancer.org/cancer/breastcancer/detailedguide/breast-cancer-classifying)^5^. The later form of breast cancer cannot be treated by endocrine therapy and is the most aggressive form of the disease^6^. In addition, the triple negative type is categorized recently into 6 subtypes based on gene expression profiles^7^. Studies have been devoted to genes mutated in those genetically pre-disposed to breast cancer (e.g. BRCA1/2 and others)^8^,^9^,^10^,^11^, as well as other factors like family history^12^, ethnicity^13^, obesity^14^, breast tissue density^15^, gender^16^ environmental factors^17^,^18^ and factors related to lifestyle^19^ that play a major role in the development and progression of these cancers. However, less emphasis has been devoted to determining the association of viruses and other microorganisms with breast cancer. Interestingly, several studies with breast cancer have shown an association with herpesviruses, polyomaviruses, papillomaviruses and retroviruses^10^.

To rapidly screen many tumor samples for associated viruses and microorganisms we developed a microarray-based approach (PathoChip) containing probe sets for parallel DNA and RNA detection of viruses and other human pathogenic microorganisms^20^. The current version of the PathoChip contains 60,000 probes representing all known viruses, 250 helminths, 130 protozoa, 360 fungi and 320 bacteria^20^. The array contains two types of probes: unique probes for each virus and microorganism, and conserved probes which target genomic regions that are conserved between members of a family of organisms, thereby providing a means for detection of previously uncharacterized members of the family^20^. The PathoChip screening technology includes an amplification step that allows detection of microorganisms and viruses present in low genomic copy number in samples. Thus the PathoChip technology has increased sensitivity relative to other microbiome screening assays, and wider coverage across kingdoms^20^. This allows multiple tumor samples to be rapidly and sensitively screened for the presence of microbial agents.

In the present study, we used the PathoChip technology to screen 100 triple negative breast cancer (TNBC) samples as well as 17 matched and 20 non-matched controls. We have identified probes which represent viruses and other microorganisms significantly detected in the breast cancer samples compared to controls. These probes were used for both PCR verification, and as capture reagents on magnetic beads to select hybridizing sequences from the breast cancer samples, which were sequenced by MiSeq for additional verification. The data establish unique microbial signatures for triple negative breast cancer with implications for future diagnostic development as well as therapeutic interventions for TNBC.

## Results

### PathoChip screening of TNBC samples detected signatures of viruses and other pathogenic microorganisms

**Figure 1:**
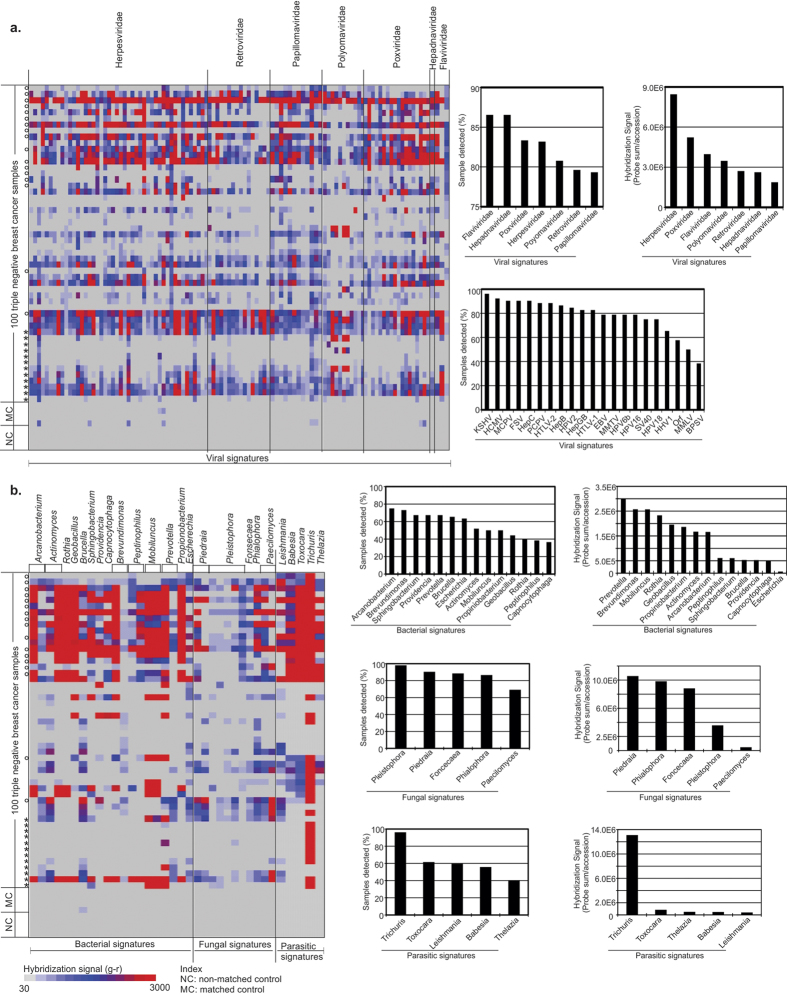
Detection of viral and microbial signatures associated with triple negative breast cancer samples. Signals for conserved and specific viral probes (**a**), bacterial, fungal and parasitic probes (**b**) detected in the triple negative breast tumor samples are shown as heat maps of probes (x-axis) hybridized to the tumor samples and both matched (MC) and non-matched control (NC) samples (y-axis). The 60 cancer samples that were pooled as 5 samples in one reaction are marked with an asterisk (*), the 40 other cancer samples were tested individually. The matched controls were obtained from the adjacent normal breast tissues of the breast cancer samples marked as zero (o). The percentage detection of conserved and specific viral signatures (**a**) and specific microbial signatures (**b**) in 100 triple negative breast tumors are shown as bar graphs. The signatures of viral family and microbes detected are ranked according to prevalence (samples detected %) and also according to decreasing hybridization signal of the probes to the tumors.

We screened 100 TNBC samples along with 17 matched, and 20 non-matched controls using the PathoChip. All samples were derived from formalin-fixed paraffin embedded archival samples (see Methods). Of the 100 TNBC samples screened, 40 were screened individually and 60 were screened in pools of 5 samples (10ng each of RNA/DNA) per reaction, so a total of 52 arrays were used to screen the 100 triple negative cancer samples. From the 17 matched and 20 non-matched controls, samples were pooled to have 4 arrays each for screening the matched and non-matched controls. Signals g-r (Cy3-Cy5) was normalized for each of the 60,000 probes for the test samples and control arrays. Normalized signals which were positive in the controls were then compared to the test samples to determine the probes that were unique to the test samples with significantly higher signals. The results of these are represented by the heat maps which are shown. The results detected viral conserved and specific probes, as well as bacterial, fungal and parasitic probes in cancer samples (Fig. 1 and Table 1 & 2). A probe was considered positive when the PathoChip screen detected a higher Cy3 (g) signal than Cy5 (r) signal for a particular probe and probes of a particular organism was known to be associated with cancer samples when the detectable hybridization signal (g-r>30) was found significantly higher in cancer samples compared to matched or non-matched control samples (Supplementary Fig. S1, Supplementary Table S1). The viral, bacterial, fungal and parasitic signatures detected in the triple negative breast cancer samples were found to be significantly associated with the cancer samples (*p* < *0.05*) compared to the non-matched and matched control samples analyzed. The *p*- values for the association of the candidate organisms as determined by the probe signals in the cancer versus the control tissues are provided in Supplementary Table S1. Two different kinds of probe sets for viruses are contained on the PathoChip^20^. The first are specific probes which are designed to detect a specific virus, for example probes that would detect Human Cytomegalovirus over all other herpesviruses or Merkel cell polyomavirus over all other polyoma viruses. The second set are conserved probes which represent sequences that are highly conserved between members of a family of viruses, for example sequences conserved between all herpesviruses. The purpose for the conserved probes is to be able to detect heretofore unknown members of a family, for example a new human herpesvirus.

The probes of a candidate organism detected by the TNBC samples showed a wide range of hybridization signals across tumor samples (Supplementary Fig. S1). We report here the percentage of samples that had detectable hybridization signal (g-r>30) for each probe of an organism without differentiation of high or low signal. Additionally, we list the names of specific viruses and microorganisms that were detected by specific probes on the PathoChip. However, we note that the detection by specific probes may suggest a closely related family member and not the specific organism. This is particularly relevant in cases where TNBC samples showed a range of hybridization signals, or no hybridization signals for some probes across the probe set for a specific virus or microorganism. It could also mean that genomic regions of these agents are deleted in that particular tumor or a variance in a strain.

Among the conserved probes, viral signatures belonging to Herpesviridae, Retroviridae, Parapoxviridae, Polyomaviridae, Papillomaviridae families were detected. For the herpesviridae family, probes of Human Cytomegalovirus (HCMV), Human Herpesvirus 1 (HHV1; Herpes simplex type 1), Kaposi sarcoma herpes virus (KSHV), Epstein-Barr virus or Human Herpesvirus 4 (EBV/HHV4) were significantly detected among 92%, 65%, 96% and 78% of the breast cancer samples, respectively (Fig. 1a, Supplementary Table S1). In the Poxviridae family, conserved probes for the parapoxviruses were significantly detected (p < 0.05) in 83% of the triple negative breast cancer samples (Fig. 1, Supplementary Table S1). Among the retroviruses, specific probes of Fujinami Sarcoma virus (FSV) and Mouse mammary tumor virus (MMTV) were detected in 90.4% and 78.8% of the breast cancer samples, respectively (Fig. 1a). Among the Polyomaviruses, specific probes detected signatures for Merkel cell Polyomavirus (MCPV) and Simian Virus 40 (SV40) in 90.3% and 75% of the breast cancer samples, respectively (Fig. 1a). For the papillomavirus family, specific probes detected Human Papilloma virus (HPV)6b, HPV18, HPV2 and HPV16 in 78.8%, 75%, 84.6%, and 78.8% of the breast cancer samples, respectively (Fig. 1a). Specific probes also detected signals for Hepatitis GB, C and B in 82.7%, 90.4%, and 86.5% of the cancer samples, respectively (Fig. 1a). Interestingly, not all the specific probes of these viral agents were detected (Table 1). This can be due to a number of possibilities which include a similar organism with identical sequence for the probe region, fragments of an organism being present or integrated fragments of organismal DNA. Studies to determine the likely possibility are ongoing.

The viral probes detected, when ranked according to percent prevalence (regardless of hybridization intensity) showed signatures of Hepadnaviruses and Flaviviruses (86.5%), followed by Parapoxviruses (83.3%), Herpesviruses (83.2%), Retroviruses (79.6%), and Papillomaviruses (79.3%). However, when ranked according to decreasing hybridization signal (the total hybridization signal of individual probes per organism, i.e Probe Sum/Accession), Herpesvirus probes had the highest hybridization signal across the tumors, followed by high hybridization signal for the probes of Parapoxviruses, Flaviviruses, Polyomaviruses, Retroviruses, Hepadnaviruses and Papillomaviruses (Fig. 1a, Table 2).

The bacterial signatures were detected in triple negative breast cancer samples and were ranked according to percent prevalence (Fig. 1b). For the bacterial signatures detected (Fig. 1b and Tables 1 and 2), the highest prevalence was of probes to detect *Arcanobacterium* (75%), followed by probes detecting the 16S rRNA signatures of *Brevundimonas, Sphingobacteria, Providencia, Prevotella, Brucella, Eschherichia, Actinomyces, Mobiluncus, Propiniobacteria, Geobacillus, Rothia, Peptinophilus,* and *Capnocytophaga* (Fig. 1b). The bacterial probes of *Prevotella* showed the highest hybridization signal, followed by very high hybridization signals for probes of *Brevundimonas, Mobiluncus, Rothia, Geobacillus, Propiniobacteria, Actinomyces* and *Arcanobacterium*; moderate hybridization signal for probes of *Peptinophilus, Sphingobacteria, Brucella, Providencia and Capnocytophaga* and low hybridization signal for probes of *Escherichia*.

The fungal signatures were of rRNA probes that recognize *Pleistophora* which were detected in 98% of the breast cancer samples, followed by probes of *Piedra, Foncecaea, Phialophora and Paecilomyces* (Fig. 1b, Table 2). The highest hybridization signal was seen for the probes of *Piedra*, followed by high hybridization signal in probes for *Phialophora, Foncecaea* and *Pleistophora* and moderate hybridization signal for probes of *Paecilomyces* (Fig. 1b).

Probes detecting the parasitic signatures of *Trichuris* were detected in 96% of the triple negative breast cancer samples, followed by *Toxocara, Leishmania, Babesia* and *Thelazia* (Fig. 1b, Table 2). Based on the ranking of hybridization signal, probes of *Trichuris* showed the highest hybridization signal, followed by high hybridization signal for probes of *Toxocara* and moderate hybridization signal for *Thelazia, Babesia* and *Leishmania*.

### Hierarchical Clustering reveals two distinct microbial signatures in TNBC samples

**Figure 2:**
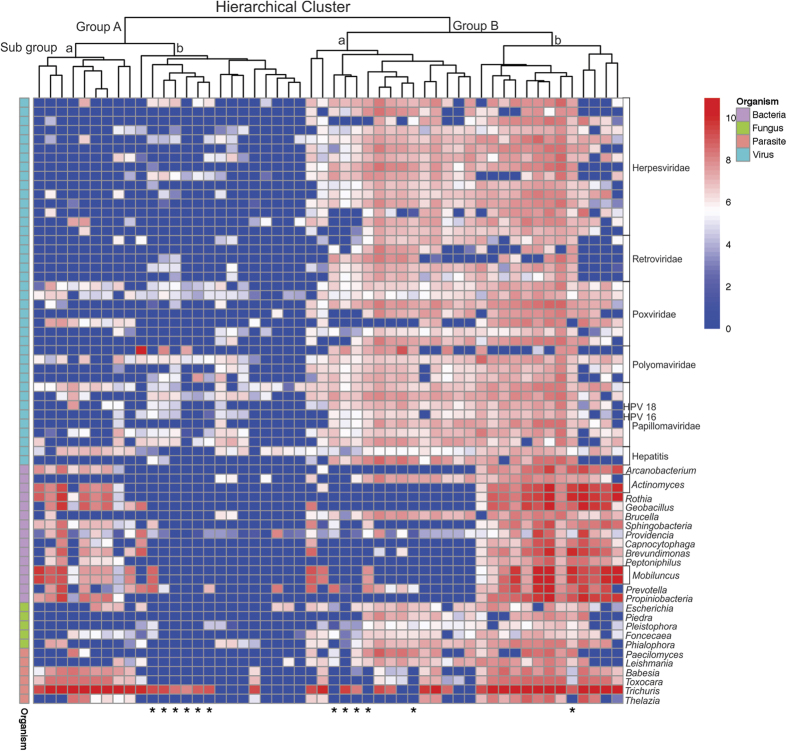
Hierarchial clustering of chosen candidate infectious agents in triple negative breast cancer samples. The 60 samples that were screened by pooling 5 samples into 1 reaction are marked with an asterisk (*). The figure shows the grouping of samples based on similar viral, bacterial, fungal and parasitic candidate signature detected in the samples.

To determine if there were similarities in detection within tumor samples hierarchical clustering of the results of screening the 100 breast cancer samples were performed. This clustered the samples into two broad groups (Fig. 2). Group B showed strong hybridization signals (red) for probes detecting viruses and fungi compared to group A TNBC samples. The group B TNBC samples were further categorized based on signals for bacteria and parasitic agents, which was found to be low (blue) in subgroup a and higher (red) in subgroup b. Within the group A TNBC samples, some samples (subgroup a) had higher detection of probes for bacteria and parasites than others (subgroup b). Notably, probes for the parasite *Trichuris* was detected in almost all the TNBC samples screened. However, the phenotypic reason for the two distinct signatures was not immediately clear since the TNBC samples tested were de-identified.

### PCR validation of signatures detected by PathoChip

**Figure 3:**
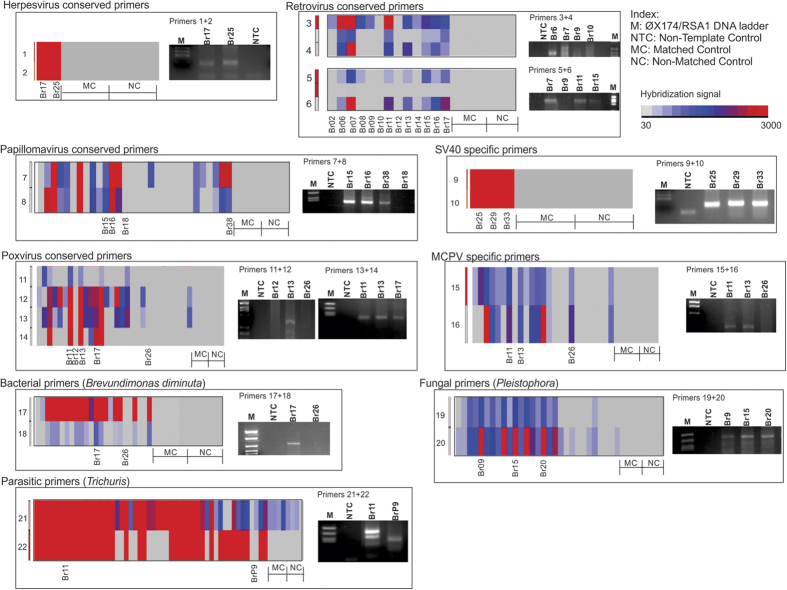
Validation of PathoChip hybridization results by PCR. Primers for PCR amplification were designed from the conserved and specific probes that hybridized to the targets used in the PathoChip screen. The heat map across the cancer and control samples for the probes from which the PCR primers were designed are shown in the left panel for each of the PCR amplification gel picture. Amplified PCR product validated the PathoChip hybridization results. MC: matched control (adjacent non-cancerous breast tissue from breast cancer patients); NC: non-matched control (breast tissue from healthy individuals). Non-template control (NTC) using sterile water was used to rule out any contamination in the PCR reaction.

PCR primers for several viruses, as well as a prevalent bacterium (*Brevundimonas*), fungus (*Pleistophora*) and parasite (*Trichuris*), were designed based on sequences from the conserved and specific PathoChip probes which showed moderate to high hybridization signals in the screen for these microorganisms. As an example of these data consider the papillomavirus conserved primers 7 and 8 which were designed from the conserved probes of papillomaviruses which showed significant hybridization for many of the samples. The PCR results show the expected amplicons for samples Br15, Br16 and Br38 which were positive for those papillomavirus probes in the screen. Conversely, sample Br18 was negative for these probes in the PathoChip screen and and as expected was also negative by PCR (Fig. 3). In all the cases tested (Fig. 3), the PCR amplification showed the expected amplicons for the PathoChip-detected viruses, as well as the selected bacterium, fungus and parasite (Fig. 3). Sequencing of the PCR products verified the detection of the appropriate virus or other microorganism. Likewise, the samples that were negative by PathoChip screens for a particular virus or organism were negative by PCR analysis. These data validate the results from the PathoChip screen supporting the presence of these microorganisms in TNBC samples.

### Probe capture for target sequencing to identify the signature organisms associated with triple negative breast cancer

**Figure 4:**
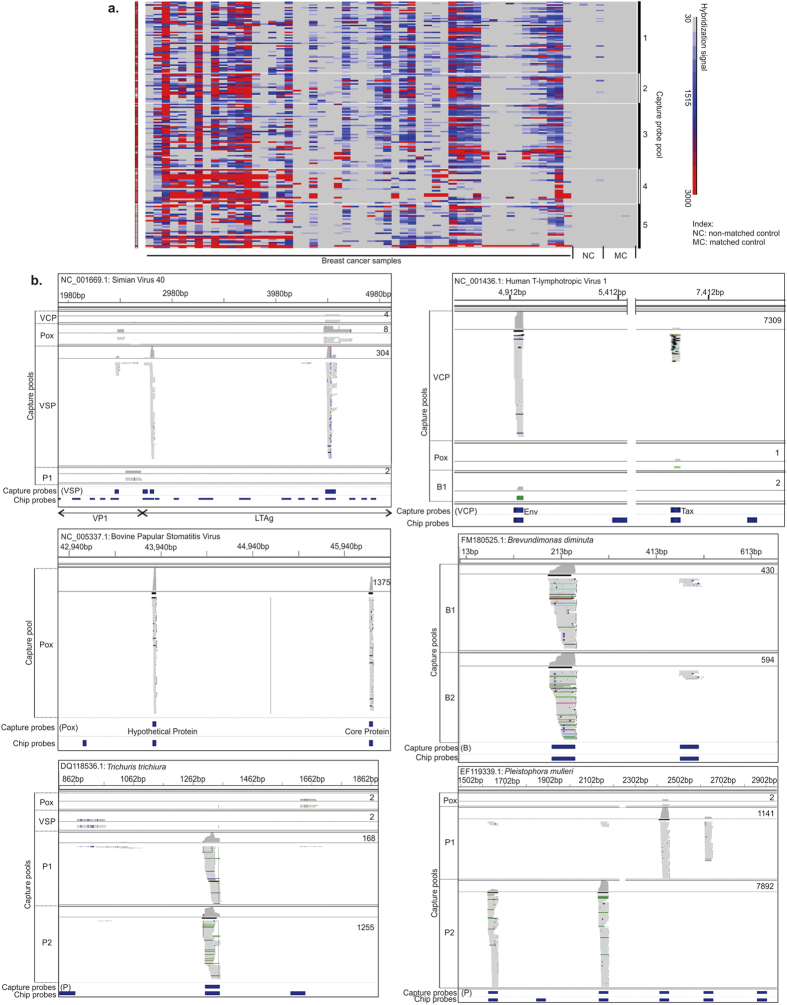
Capture pool used for nucleic acid capture and MiSeq analysis. (**a**) The heat map indicates test minus reference signals from the probes (Y-axis) chosen from 4 different analyses. 7 separate captures of target nucleic acids were done using 5 probe pools as indicated. (**b**) The individual reads obtained from MiSeq are shown for the triple negative breast cancer samples. Whole genome amplified DNA plus cDNA was hybridized to a set of biotinylated conserved and specific viral, bacterial, fungal, parasitic and viroid probes, captured on Streptavidin beads, and used for tagmentation library preparation and deep sequencing with paired-end 250-nt reads. The MiSeq was done on libraries generated by capture sequences using viral conserved probes (probe pool VCP), viral specific probes (probe pool VSP), pox virus probes (probe pool Pox), bacterial probes (probe pool B1 and B2), fungal/parasitic and viroid probes (probe pool P1 and P2). The MiSeq reads when aligned with the metagenome of PathoChip (Chip probes) clustered mostly at the probe regions of the represented organisms. The genomic location along with the number of MiSeq reads are shown on the figure and represents the genomic co-ordinates. The alignment track of IGV displays two tracks: (the upper) a coverage track and (the lower) the alignment track. IGV colors paired-end alignments that deviate from expectations (horizontal colored lines) and the mismatched bases are displayed in color (A as green, C as blue, G as yellow and T as red) on the grey aligned sequence bar that represents the read.

For additional validation of the PathoChip detection of viruses, bacteria, fungi and parasites in the TNBC samples, probes with stronger hybridization signal with the breast cancer samples and not in the controls were selected for target capture and sequencing. Hybridization signals for those probes across all the triple negative breast cancer, matched and non-matched controls analyzed in the study are presented as a heat map in Fig. 4a. Five probe pools (probe pool 1–5) were used to capture the targets from the pooled samples. Seven target capture reactions were done with the 5 probe pools (Fig. 4) described in the methods section [Viral Conserved Probe (VCP) capture, Pox capture, Viral Specific Probe (VSP) capture, Bacterial probe captures (B1 and B2) and Fungal/Parasitic/Viroid Probe captures (P1 and P2)]. The seven captured targets sequencing libraries were made, pooled and sequenced using MiSeq. The MiSeq data were aligned with the PathoChip metagenome. The data showed that the MiSeq reads clustered, in large part, around the genomic locations of the probes used in the capture reactions; although occasionally regions of the target genomes outside the locations of the probe were detected (Fig. 4b). The number of MiSeq reads of the candidate organisms for each capture is shown in Supplementary Table S5 and Supplementary Fig. S2.

#### Viral Genomes.

The MiSeq reads confirmed the presence of viral genomic regions of polyoma viruses (SV40, JC, MCPV); herpesviruses (HCMV); papilloma viruses (HPV16, HPV18, HPV2); retroviruses (HTLV1, MMTV), poxviruses (Pseudo cowpox virus, Bovine papular stomatitis virus and Orf virus) (Supplementary Fig. S2).

One of the most prevalent MiSeq reads (9669) aligned to a non-coding regulatory region of JC polyomavirus and was selected by a virus conserved probe (VCP) capture. In addition, target capture using specific probes of SV40 and MCPV revealed 304 and 1375 MiSeq reads that mapped to the large T-antigen genes of SV40 and MCVP, respectively. These data support the association of a polyoma-like virus with triple negative breast cancer. VCP capture also resulted in 2,552 MiSeq reads which mapped to UL70 (primase) and UL104 (capsid) of HCMV, and the specific probe capture yielded 382 reads that mapped to the HCMV non-coding RNA 4.9, as well as the UL77 and UL98 genes. Specific probes capture resulted in 670 reads which aligned to the E2, E4 and L2 region of HPV16 genome and 99 reads that aligned to the L1 region of HPV18 genome. Additionally, HPV-2 sequences were indicated by 86 reads aligned to HPV-2 E1 as well as the genomic sequences between the HPV-2 E4 and L2 genes. Hepatitis viral genomes were indicated by 111 reads that aligned with the probe sequence within the E1/E2 polyprotein and the non-structural 5A genomic sequence of the Hepatitis C genotype 1. 96 reads aligned with the probe corresponding to the S protein of Hepatitis B. Retroviral genomes were detected by VCP capture where 7,319 reads aligned to the Rex/Tax and env genes of HTLV-1; and 33 and 78 reads from the VCP and VSP mapped to the p140 polyprotein gene of Fujinami sarcoma virus (FSV)^21^. Further, specific probe capture yielded 138 sequence reads that aligned to the super-antigen and pol/env genes of mouse mammary tumor virus^21^. Poxviral genomic regions were indicated by VCP capture, where 637 reads aligned to the DNA polymerase and tyrosine phosphatase genes of pseudocowpox virus, 3,277 reads aligned to the ORF041 (hypothetical protein), the ORF044 (core protein) and ORF064 (mRNA capping enzyme large sub-unit) of the Bovine Papular Stomatitis Virus, and 588 reads aligned to the hypothetical protein encoding gene of Orf virus.

#### Bacterial genomes.

Specific bacterial probes used for target capture and sequencing resulted in MiSeq reads that aligned to the 16S rRNA genomic locations of the bacterial signatures that were detected by the PathoChip screen; namely, *Brevundimonas diminuta, Arcanobacterium haemolyticum, Peptoniphilus indolicus, Prevotella nigrescens, Propiniobacterium jensenii and Capnocytophaga canimorsus* (Fig. 4b, Supplementary Fig. S2, Supplementary Table S5).

#### Fungal and parasite genomes.

The fungal and parasitic pooled probes (P) captured targets that mapped to rRNA genes of the following fungal organisms: *Pleistophora mulleris, Piedraia hortae, Paecilomyces reniformis, Phialophora verrucosa* and *Fonsecaea pedrosoi;* and the 18S rRNA regions following parasites: *Trichuris trichura, Thelazia gulosa* and *Leishmania major* (Fig. 4b, Supplementary Fig. S2, Supplementary Table S5).

In sum, the targeted probe capture and sequencing data support the results of the PathoChip screen suggesting that genomic signatures for the detected viruses, other microorganisms, or their closely related family members, are much more frequently associated with TNBC tissues than normal tissues.

## Discussions

In the present study, we detected predominant viral, bacterial, fungal and parasitic genomic sequences in 100 triple negative breast cancer samples using the PathoChip array which contains a set of 60,000 probes that cover all known viral agents as well as human pathogenic bacterial, fungi and parasites^20^. Using this sensitive approach we detected multiple viruses and other microorganisms in triple negative breast cancer samples. These results were validated by PCR and target capture sequencing. Hierarchical analysis shows that at least two major microbial signatures can be found within the TNBC samples tested with minor subgroups within the two major groups. It is important to point out that our data at this time only tell us that these viruses and other microbial agents can be associated with the tumor tissue or tumor micro-environment. The data currently do not implicate these viruses and microorganisms as causative or contributory to the development of TNBC. While they could contribute, it is also possible that the tumor tissue and the tumor microenvironment provide an amiable niche for them to persist. In either case the presence of these viral and other microbial signatures may provide diagnostic capabilities with implications for future targeted interventions.

The PathoChip screening data are in agreement with the findings of other reports that suggest the association of viruses with a variety of cancers. For example, previous studies suggest the presence of herpesvirus, papillomavirus, polyomavirus and MMTV-like sequences in breast cancer^21^,^22^,^23^,^24^,^25^. One study reported a much higher rate of HCMV infection (97%) in biopsy specimens of breast cancer patients compared to controls by immunohistochemistry^24^. Others have reported EBV DNA from breast cancer samples by PCR and suggested the association of EBV with more severe forms of breast cancer^22^,^23^,^25^. A study examining 1,535 cases, showed significant association of EBV with increased breast cancer risk^26^. SV40 DNA sequence from the T antigen open reading frame were reported in 22% of 109 breast cancer samples as determined by PCR with confirmation by immunohistochemistry^22^. Furthermore, JCV another polyomavirus, was detected in 23% of 123 breast cancer cases by PCR^27^. Additionally, the association of HPV with breast cancer has been suggested to be up to 86%^28^. A recent study detected HPV in 15% of triple negative breast cancer patients (40 cases) but not in 40 non-triple negative cases by PCR^29^. The most frequent genotype detected was HPV-16 (28.6%), and others were HPV-31, -45, 52, -6, -66^29^.

Other studies have proposed an association between the beta-retrovirus human mammary tumor virus (HMTV) and breast cancer. This is due to the detection of MMTV-like sequences in breast cancer samples and not in normal tissues^30^; Of importance to note that HMTV has 95% sequence homology with MMTV^31^. The env, gag and sag HMTV gene sequences from patients with breast cancer have been cloned and sequenced suggesting the existence of this virus or a close relative in breast cancer patients^32^. That multiple viruses can co-exist in the same breast cancer sample has been suggested by studies showing the presence and co-existence of EBV (68%), HPV (50%) and MMTV (78%)^22^. In sum these data suggest a substantial presence of viruses in tumor tissue. Our PathoChip screen of TNBC suggests that many of these viral signatures can be associated with one specific cancer, TNBC, along with the presence of signatures for bacteria, parasites and fungi. These signatures can potentially be co-operating in the micro-environment for their own commensalistic existence or may also have contributory roles in driving the pathogenic process or a combination of both of these possibilities. Regardless, this provides us with the possibility of utilizing this signature pattern for diagnostic purposes which will be developed in future studies.

It should be noted that the detection of viral sequences in tumor tissues can be a challenge. Studies using RNA-Seq with billions of reads has not been strongly positive except for the previously identified prevalent viral agents in known cancers (example, cervical carcinomas, head and neck squamous cell carcinoma)^33^. Our techniques combined the sensitivity of detection with next generation sequencing (NGS) to detect extremely low copy number of an agent including its family member and so may increase the power of detection of these organisms across kingdoms^20^.

It is interesting that TNBC samples fell into hierarchical groups showing at least two distinct microbial signatures. One hierarchical group (group B) was prevalent in viruses: a herpesvirus-signature (primarily β- and γ-herpesvirus-like); a parapoxvirus signature (parapox virus family-like); flavivirus (hepatitis C- and GB-like); polyomavirus (JC- MCPV- and SV40-like); retrovirus (MMTV-, HERV-K-, HTLV-like); hepadnavirus (hepatitis B-like) and papillomavirus (HPV-2, 6b and 18-like). This hierarchical group also tended to be higher in fungal signatures and suggested representatives of the *Pleistophora, Piedraia, Fonsecaea, Phialophora* and *Paecilomyces* families. Bacterial and parasitic signatures could be found equally between the two hierarchical groups. Bacterial probes included representatives of a number of families (Actinomycetaceae, Caulobacteriaceae, Sphingobacteriaceae, Enterobacteriaceae, Prevotellaceae, Brucellaceae, Bacillaceae, Peptostreptococcaceae, Flavobacteriaceae), some of which have been associated with cancers^34^,^35^,^36^,^37^ and parasitic signatures included representatives of the *Trichuris* (highly detected in most of the TNBC samples screened), *Toxocara, Leishmania, Thelazia* and *Babesia* families. In fact, there has been one report on the association of parasites with metastatic breast cancer^38^. It is interesting that the associated viral signatures may provide clues as to a potential pathogenic role based on previous reports. The fact that there are two distinct groups based on the hierarchical analysis suggests a possible separation of TNBC based on associated microorganisms. Nevertheless, future studies characterizing these groups will be critical to provide further insights into the disease.

The PathoChip screen also provided some surprising results. For example, detection of the sequences related to Okra mosaic virus^39^, and citrus viroid V (Supplementary Fig. S1, Supplementary Fig. S2, Supplementary Table S1). A study that utilized de novo assembly of viral genome in a tumor RNA-Seq, detected sequences of Tomato Mosaic Virus in one uterine endometrial carcinoma tumor^33^. Interestingly, the detection of RNA for viroids is supported by a study which suggested intra-nuclear viroids in breast cancer^40^. Additionally, dietary raw fruits and vegetables expose us to large numbers of plant viruses and viriods, some of which may persist. The screen also detected genomic sequences similar to a baculovirus. We cannot be sure why sequences related to insects and plant viruses were detected but it is quite possible that variants can persist in human under specific situations. We note that the screen may be biased toward DNA viruses since RNA viral genomes are more prone to degradation in FFPE samples. However, at this point the data suggest that a microbial signature can be delineated in TNBC and that this signature is underrepresented in normal tissue. Further characterization of TNBC samples as well as samples from other cancers will reveal if this signature represents a TNBC-specific signature or a general signature for cancer. However, the data is certainly intriguing and challenges our norms as to the role of microorganisms in cancer, as well as determining their interactions with the tumor tissue or other microorganisms in the micro-environment. The sensitivity of the Pathochip combined with NGS provides a powerful tool for future studies in this arena and is ongoing.

## Methods

### PathoChip design

The metagenomic approach for the design of the 60,000 probe sets of selected microorganisms used on the PathoChip Array has been previously described^20^. The designed probe sets were manufactured as SurePrint glass slide microarrays (Agilent Technologies Inc.). Probes were represented as 60-nt DNA oligomers with 60,000 probes on 8 replicate arrays per slide^20^. These target pathogenic viral, prokaryotic, and eukaryotic genomes with multiple probes for each organism is combined with upstream sample preparation and amplification protocols to detect DNA and RNA of microorganisms and downstream data analysis. PathoChip screening of DNA plus RNA from formalin-fixed paraffin-embedded (FFPE) tumor tissues has been established^20^, and the detection of oncogenic viruses was previously validated^20^. Our previous studies demonstrated the use of the PathoChip technology, combined with PCR and HT sequencing, as a valuable strategy for detecting the presence of pathogens in human cancers and other diseases^20^.

### Sample preparation and Microarray processing

100 de-identified FFPE triple negative breast cancer samples were received from the Abramson Cancer Center Tumor Tissue and Biosample Core in the form of 10 μm sections on non-charged glass slides and 17 matched and 20 non-matched control samples were provided as paraffin rolls. Matched controls were obtained from the adjacent non-cancerous breast tissue of the same patient from which the cancer tissues are obtained. Non-matched controls are breast tissues obtained from healthy individuals. The rolls or mounted sections (5 sections per sample) from FFPE samples were used for parallel DNA and RNA extraction) as previously described^20^. The quality of the extracted DNA/RNA was assessed by measuring the A_260/280_ ratio. The size distributions of the extracted nucleic acids were determined by agarose gel electrophoresis. The extracted RNA and DNA samples were partially degraded as expected and were subjected to RNA/DNA amplification (Whole Transcriptome Amplification/WTA) as previously described^20^ using 50 ng each of RNA and DNA as input. Of the 100 triple negative breast cancer samples screened, 40 were screened individually and 60 were screened in pools of 5 samples (10ng each of RNA/DNA per sample) per reaction, so a total of 52 arrays were used to represent the 100 triple negative cancer samples. The 17 matched and 20 non-matched control samples were pooled, represented by 4 arrays each for screening the matched and non-matched controls. The amplification products were checked by agarose gel electrophoresis and as expected the size of the amplicons ranged from 200–400 bp for FFPE samples. 15 ng each of human reference RNA and DNA extracted from the BJAB human B cell line was also subjected to WTA. The amplified products were purified using a PCR purification kit (Qiagen, Germantown, MD, USA), and 2 μg of the amplified product from the FFPE cancer tissues was used for Cy3 labeling by the SureTag labeling kit (Agilent Technologies, Santa Clara, CA) and Cy5 labeling was performed on 2 μg of human reference cDNA/DNA amplification product as a control to determine cross-hybridization of probes to human DNA. The labelled DNA were purified and the extent of labeling was determined by A_550_ for Cy3 (green) and A_650_ for Cy5 (red). The labelled samples were hybridized to the PathoChip as described by Agilent Technologies, Santa Clara, CA. Hybridization cocktail consisting of a CGH blocking agent, hybridization buffer (as per manufacturer’s instruction), were added to the labeled test sample (Cy3) and the reference (Cy5), denatured and hybridized to the 8X arrays (PathoChip is a glass slide containing 8 arrays) in a 8-chamber gasket slide at 65 °C with rotation in an Agilent hybridization oven. Post-hybridization, the slides were washed using wash buffer and scanned using an Agilent SureScan G4900DA array scanner.

### Microarray Data Analyses and Statistical analysis

The microarray images were analyzed using Agilent Feature Extraction software; normalization and data analyses were done in the Partek Genomics Suite (Partek Inc., St. Louis, MO, USA) as previously described^20^. Model-based analysis of tiling arrays (MAT) which utilized a sliding window analysis of probe signals for each tumor; analysis at the individual probe level (both for specific and conserved probes) and at the accession level (taking account of all the probes per accession) were performed. While the outlier analysis at the individual (specific probe outlier and conserved probe outlier), or at the accession level (accession outlier) revealed probes that show higher hybridization signal in some samples, the paired t-tests with False Discovery Rate (FDR) multiple correction at the individual probe (specific probe t-test, conserved probe t-test) or at the accession level (accession t-test) revealed the probes that are significantly detected across the 100 tumor samples analyzed. We performed two-sample Wilcoxon tests to determine if cancer samples have significant detection of the candidate signature of organisms compared to the control (both matched and non-matched) samples. Hierarchial clustering of the samples based on the detection of pathogenic signatures was done using the R program (Euclidean distance, complete linkage, non-adjusted values)^41^,^42^.

### Validation of PathoChip results

PCR primers were designed from the conserved and specific probes of organisms with hybridization signals that represent a signature pattern. The PCR amplification reaction mixtures for each reaction contained 200 ng of tumor DNA and 10 pmol each of forward and reverse primers (Supplementary Table S2), 300 μM of dNTPs and 2.5 U of LongAmpTaq DNA polymerase. DNA was denatured at 94 °C for 5 min, followed by 30 cycles of 94 °C for 30 s, 48–57 °C for 30 s, and 65 °C for 20–60 s. The annealing temperature was different for different sets of primers used, mostly 5 degrees below the melting temperature of the forward and reverse primers for each set of primers. The PCR conditions for each of the primer sets are mentioned in Supplementary Table S2.

### Probe Capture and Next Generation Sequencing

Libraries of targeted sequences were captured by magnetic beads to generate libraries for next generation sequencing (NGS). Selected PathoChip probes with high hybridization signals in triple negative breast cancer samples only were synthesized as 5′-biotinylated DNA oligomers (Integrated DNA Technologies, Coralville, IA, USA), mixed as 5 capture probe pools (pools 1–5) (Fig. 4, Supplementary Table S3), and hybridized to pools of tumor samples. Pool 1 contained 52 selected viral conserved probes (VCPs) excluding the pox viral conserved probes; pool 2 contained 18 conserved pox viral probes (Pox); pool 3 contained 43 viral specific probes (VSPs); Pool 4 included 20 selected bacterial probes (B) and Pool 5 contained 28 fungal, parasitic and viroid probes (P). Targets were captured by pooling all 100 WTA products used for PathoChip screening (for VCP, Pox, VSP capture) or by pooling 100 WTA samples in two groups (group 1 comprising pool of 18 WTA samples that showed high hybridization signal to B and P probes and group 2 comprising the remaining WTA samples). Each capture probe pool was added to each target pool in reaction mixtures containing 3 M tetra-methyl ammonium chloride, 0.1% Sarkosyl, 50 mMTris-HCl, 4 mM EDTA, pH 8.0 (1XTMAC buffer). 7 individual target captures were done VCP, Pox, VSP, B1, B2, P1 and P2. The reaction mixtures were denatured (100°C for 10 mins) followed by a hybridization step (60°C for 3 hrs). Streptavidin Dynabeads (Life Technologies, Carlsbad, CA, USA) were added with continuous mixing at room temperature, followed by three washes of the captured bead-probe-target complexes in 0.30 M NaCl plus 0.030 M sodium citrate buffer (2XSSC) and three washes with 0.1 × SSC. Captured single-stranded target DNA was eluted in Tris-EDTA for library preparation and NGS.

The seven captured eluates were re-amplified by GenomePlex reactions (Sigma-Aldrich, St. Louis, MO), purified and assessed for size distribution by agarose gel electrophoresis. Seven sequencing libraries were prepared using Nextera XT sample preparation kit (Illumina, San Diego, CA, USA), according to the manufacturer protocols. The samples were submitted to the Washington University Genome Technology Access Center (St. Louis, MO) for quality control measurements, library pooling, and sequencing using an Illumina MiSeq instrument with paired-end 250-nt reads. Pre-processed raw reads were trimmed to remove low-quality ends (Phredscore  < 30). We then aligned reads against the human reference genome using Bowtie2 (sensitive-local mode)^43^. Reads that can be mapped to human genome with high quality were excluded and we aligned the remaining reads to the PathoChip metagenome, using Bowtie2 (sensitive-local mode)^43^. The total number of reads from each library, the number of reads mapping to pathogenome versus the human genome are shown in Supplementary Table S4. There were 680,534 reads from the 7 libraries that we were able to align to the PathoChip metagenome. We then considered the 202,905 reads with mapping quality score MapQ >=20 for further visualization and quantification analysis using Integrative Genomics Viewer 2.3.25^44^.

## Additional Information

**How to cite this article**: Banerjee, S. *et al.* A distinct microbiological signature associated with triple negative breast cancer. *Sci. Rep.* **5**, 15162; doi: 10.1038/srep15162 (2015).

## Supplementary Material

Supplementary Information

Supplementary Table S3

Supplementary Table S5

## Figures and Tables

**Table 1:**
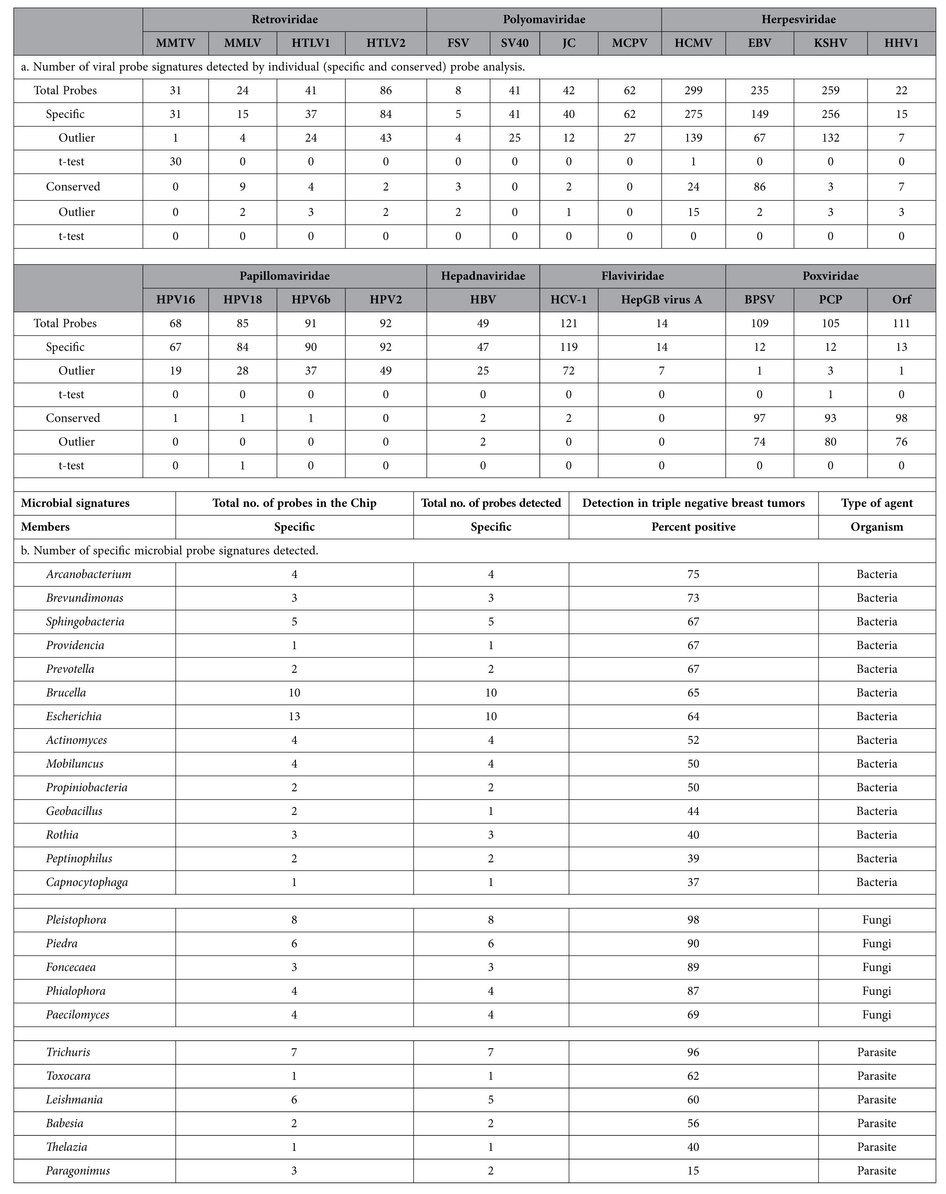
Number of viral and microbial probe signatures detected by screening triple negative breast cancer samples by the PathoChip.

**Table 2:**
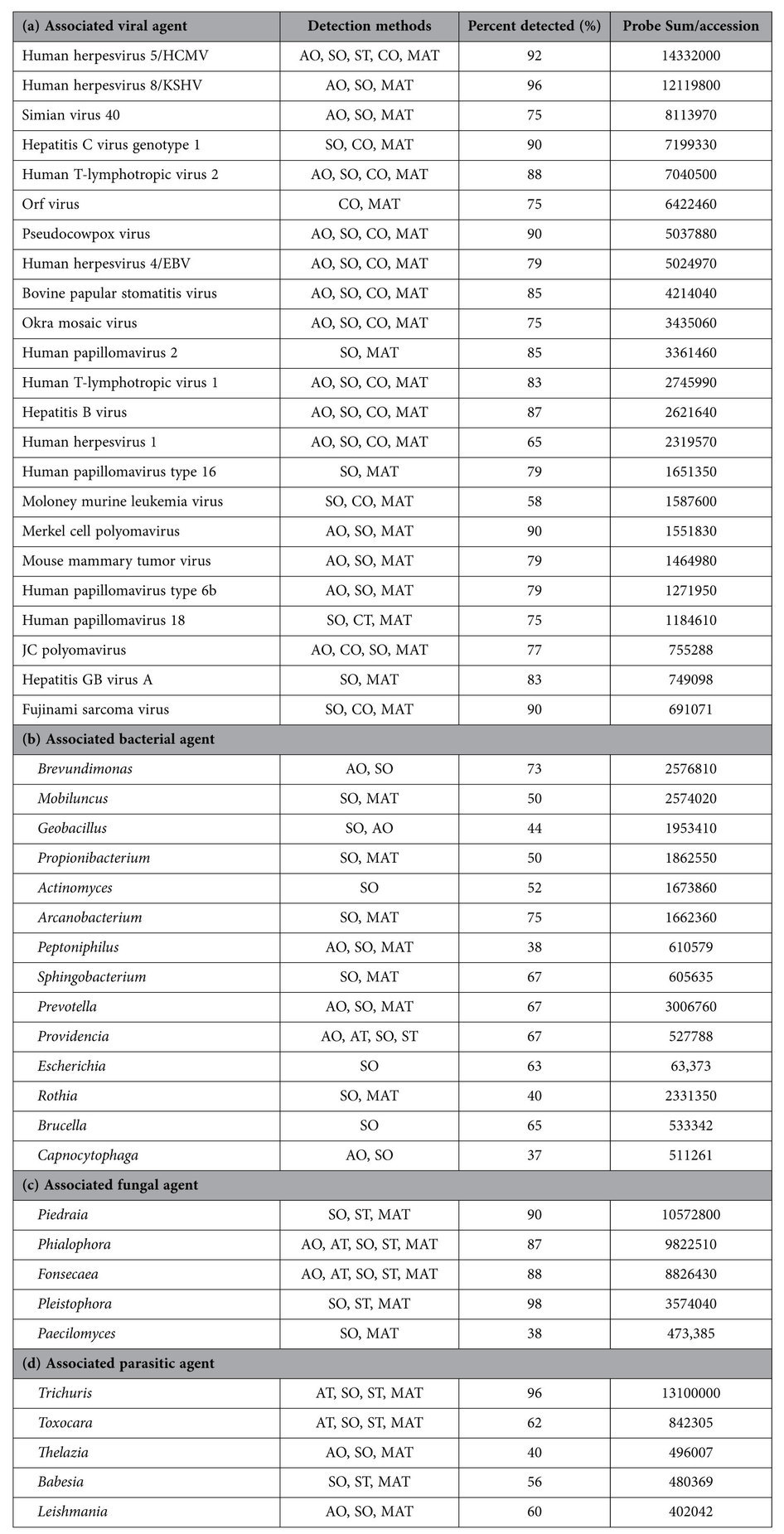
Hybridization signal (calculated as sum of hybridization signal of all the probes per accession) and prevalence of viral and microbial probes detected in 100 triple negative breast cancer samples.

The methods that detected the candidates are mentioned; AO: Accession outlier, AT: Accession t-test, SO: Specific probe outliers, ST: Specific probe t-test CO: Conserved probe outlier, CT: Conserved probe t-test, MAT: Model based analysis for tiling arrays.
