# Supplementary material for: Distinct microbiological signatures associated with triple negative breast cancer
Source: Sci Rep. 2015 Oct 15;5:15162. doi: 10.1038/srep15162 (PMC4606812; doi:10.1038/srep15162)
Supplement: Supplementary Information [file srep15162-s1.pdf]

Title: Distinct microbiological signatures associated with triple negative breast cancer

Sagarika Banerjee, Zhi Wei, Fei Tan, Kristen N Peck, Natalie Shih, Michael Feldman, Timothy Rebbeck, James Alwine, Erle S Robertson

Supplementary Figure S1
Viral probes.

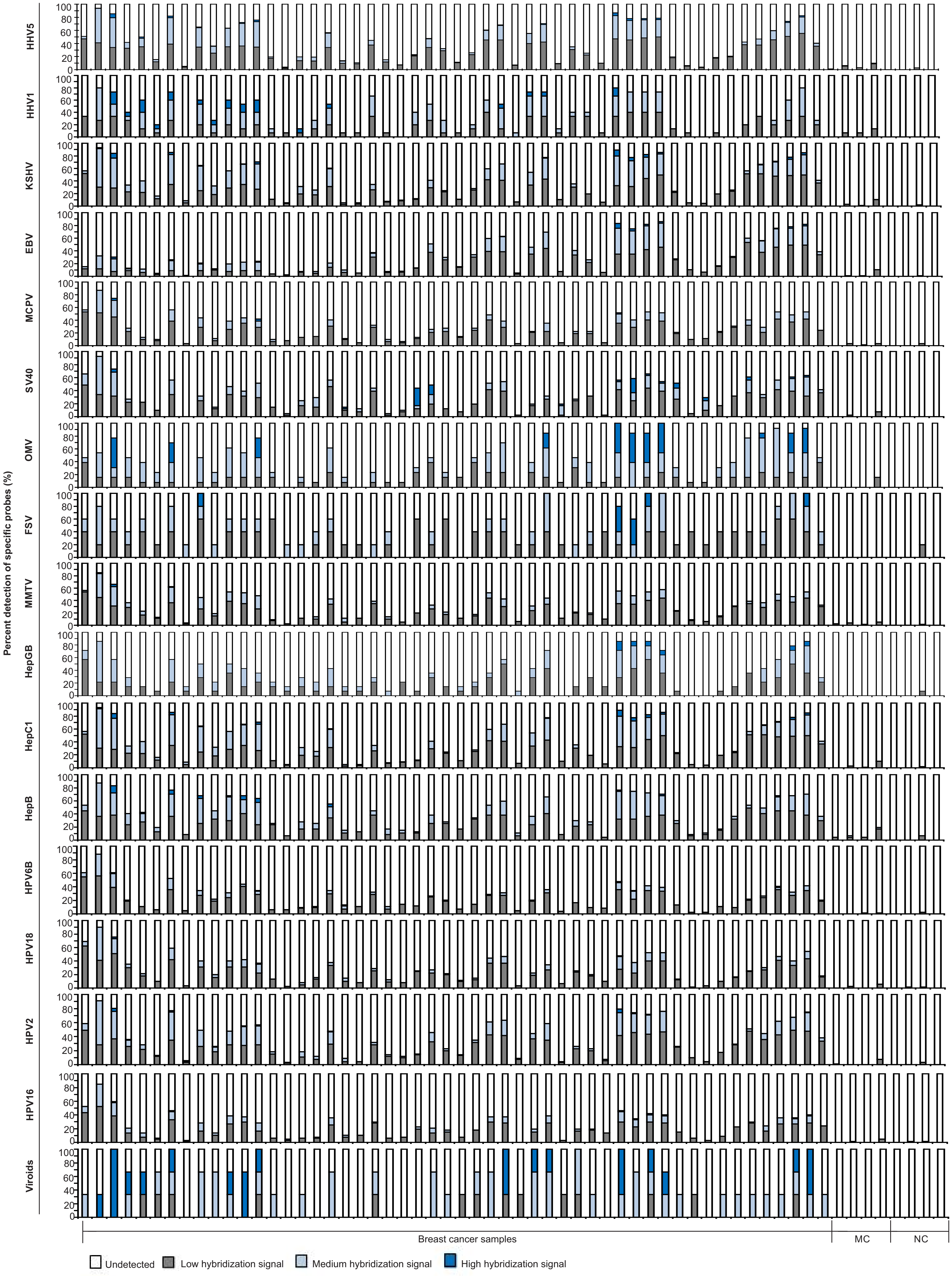

Supplementary Figure S1
Bacterial probes.

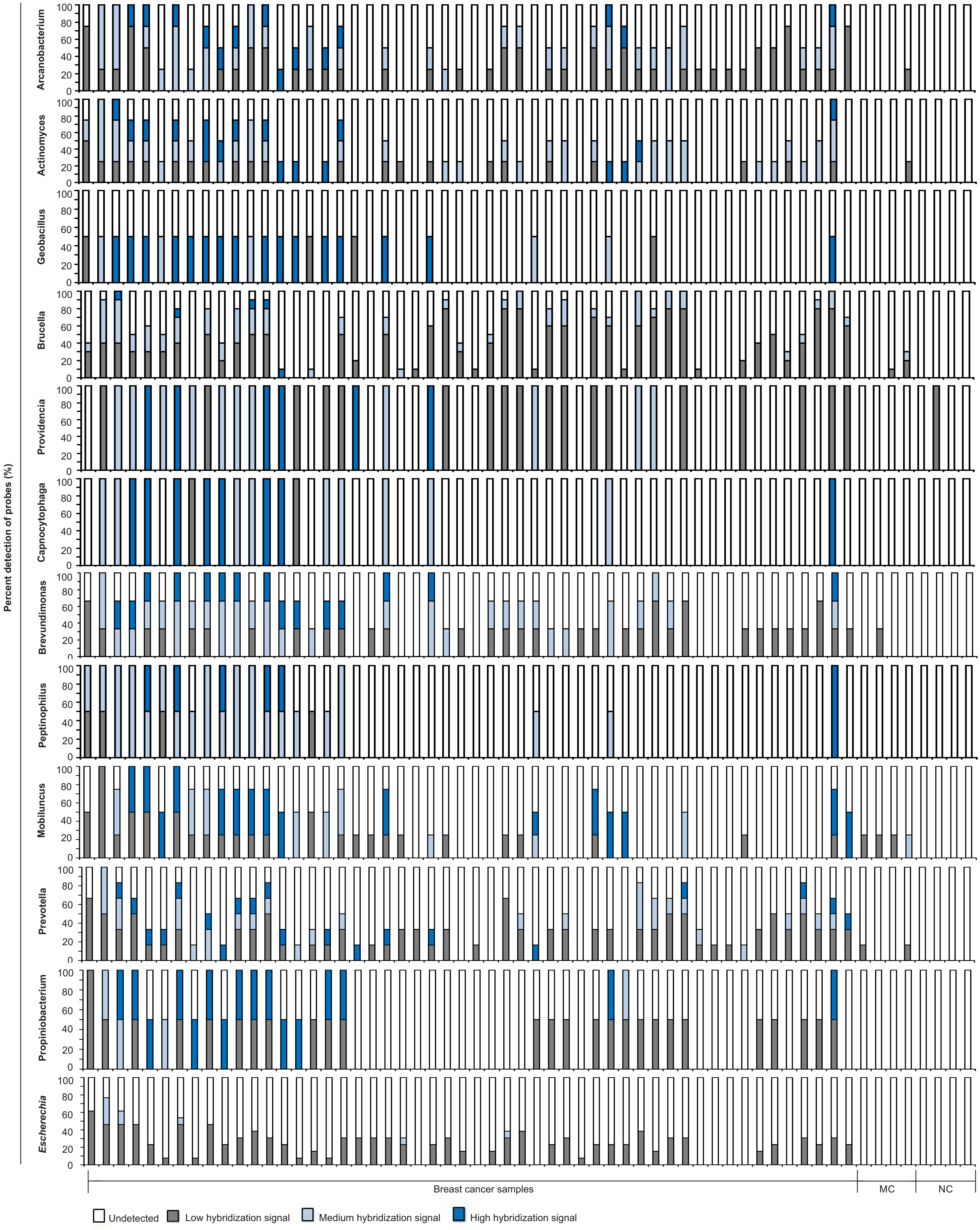

Supplementary Figure S1

Fungal probes.

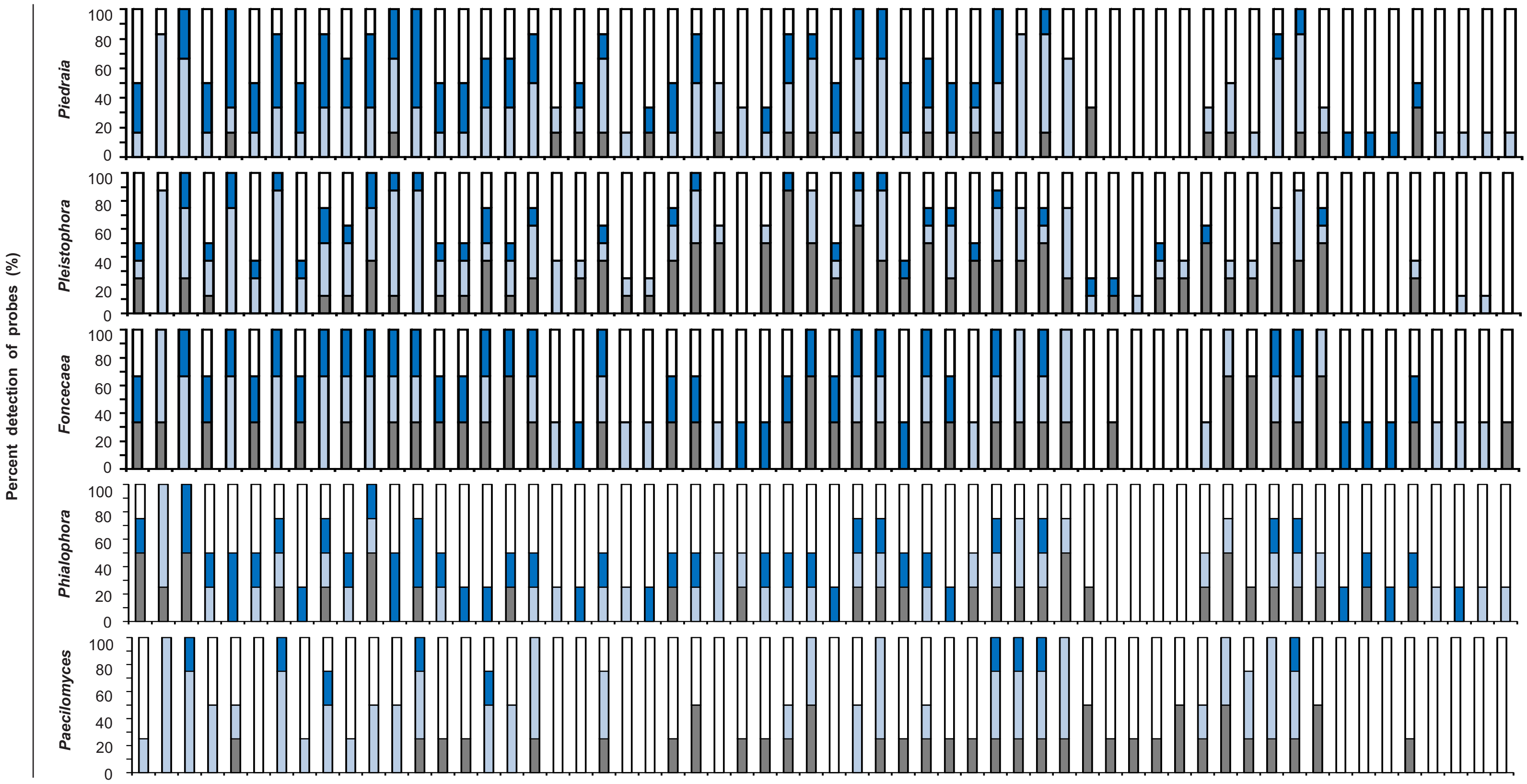

Parasitic probes.

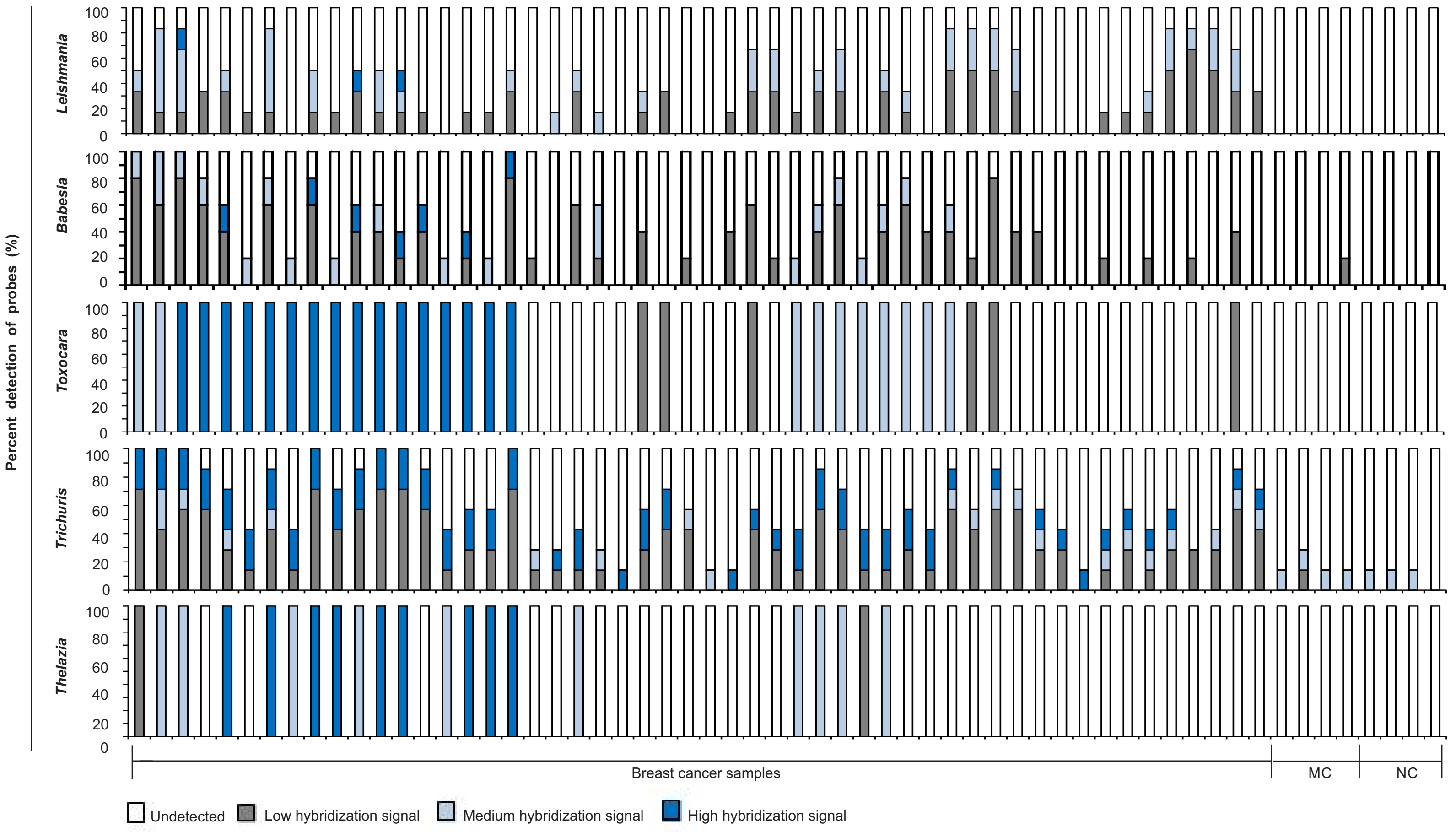

Supplementary Figure S2

Viral Signatures

Herpesviridae

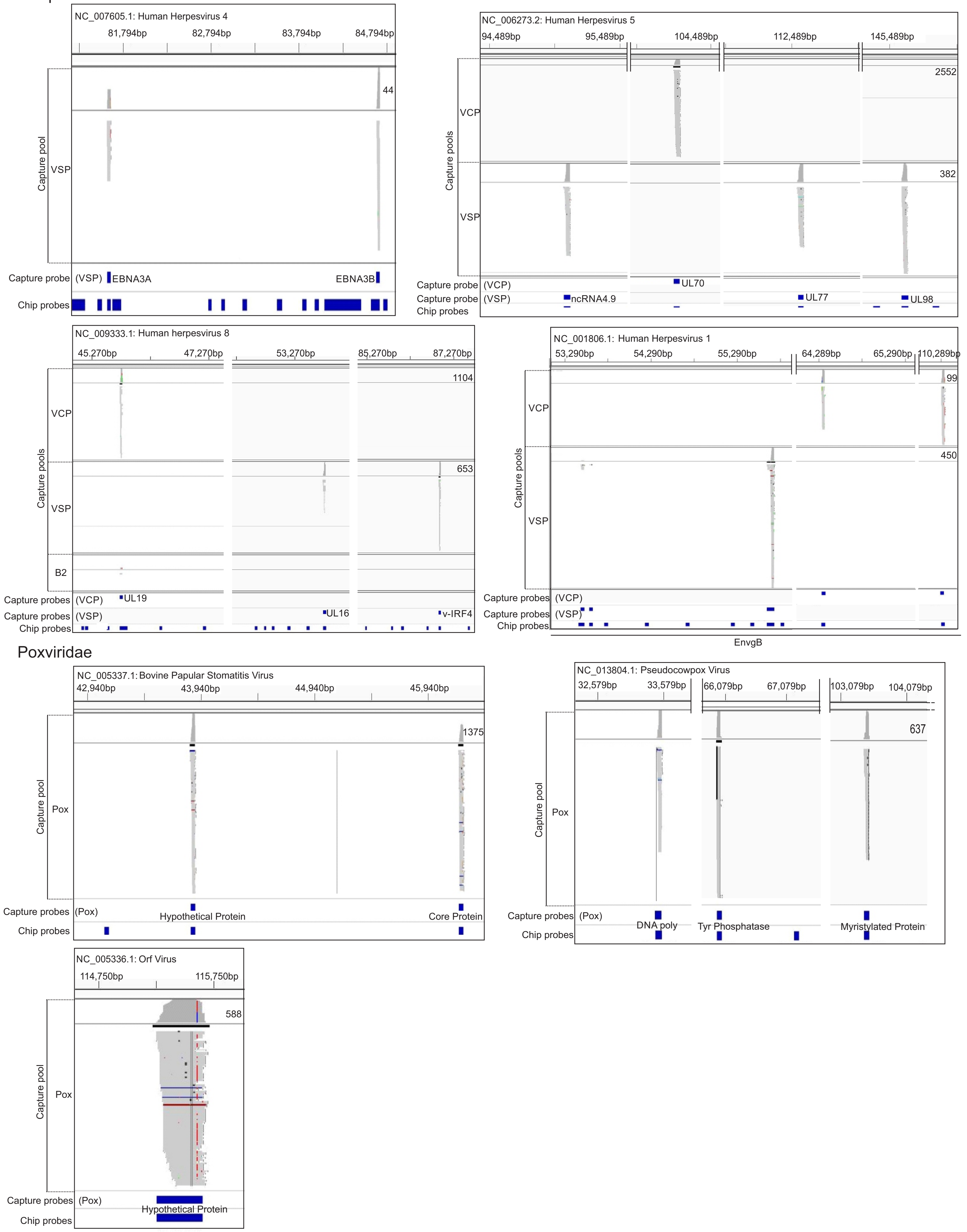

Supplementary Figure S2

Viral Signatures

Retroviridae

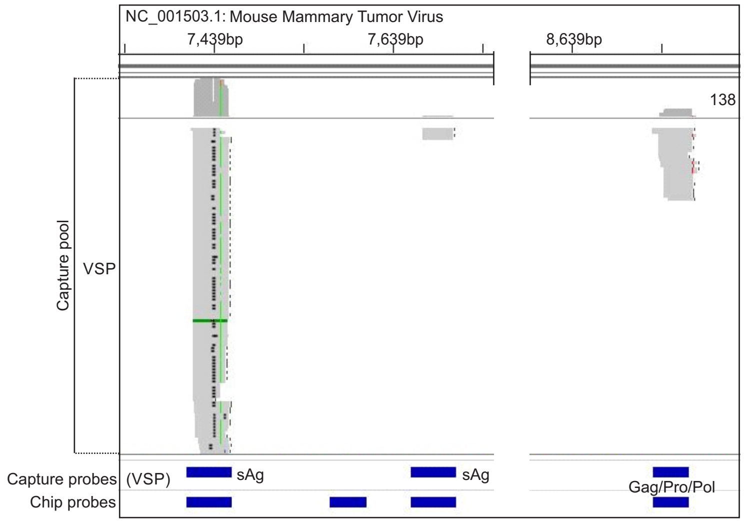

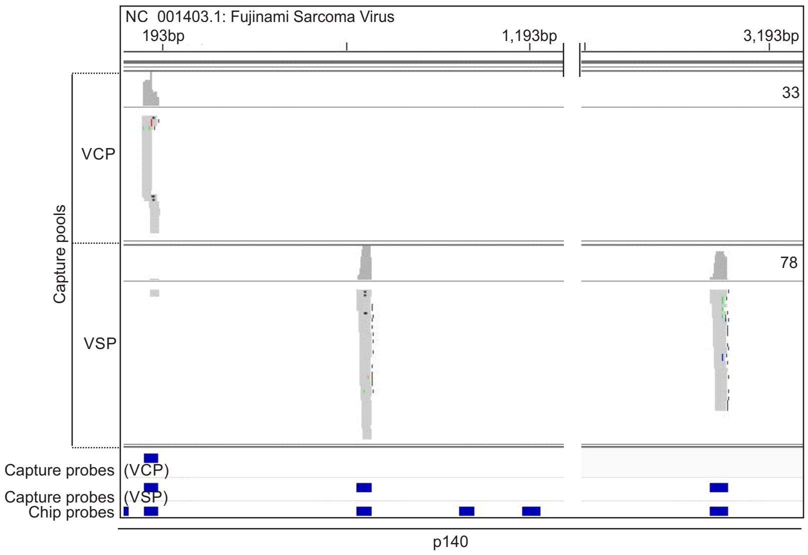

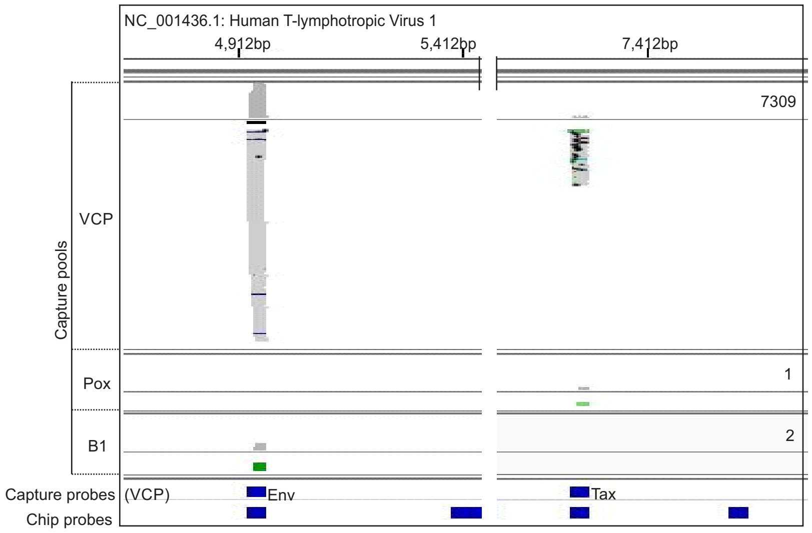

Flaviviridae

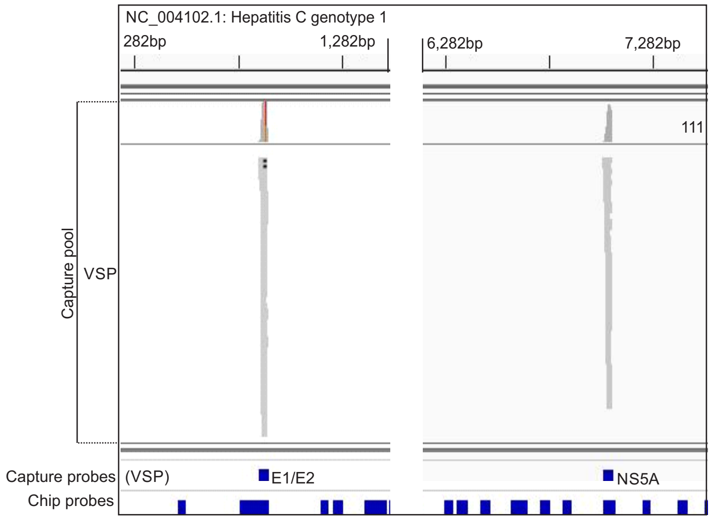

Hepadnaviridae

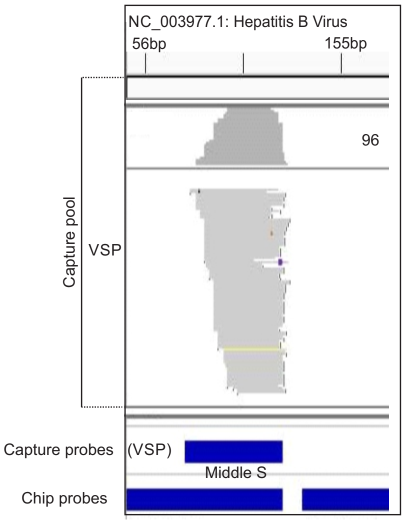

Supplementary Figure S2

Viral Signatures

Polyomaviridae

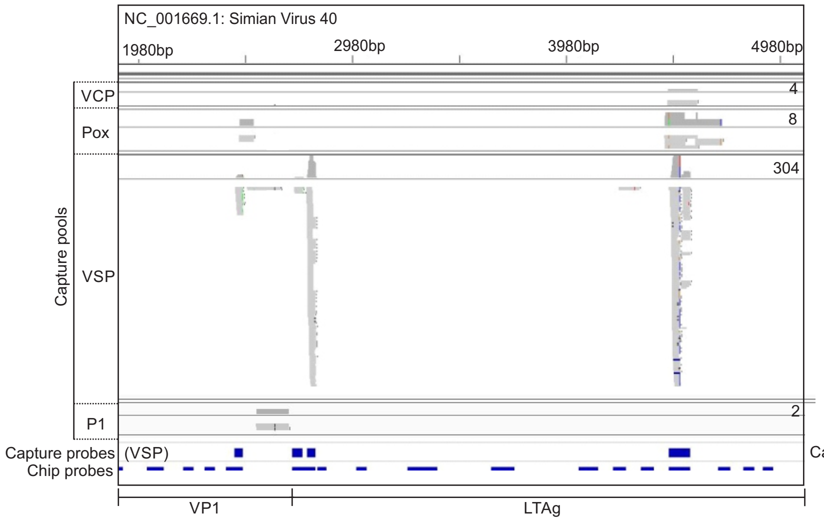

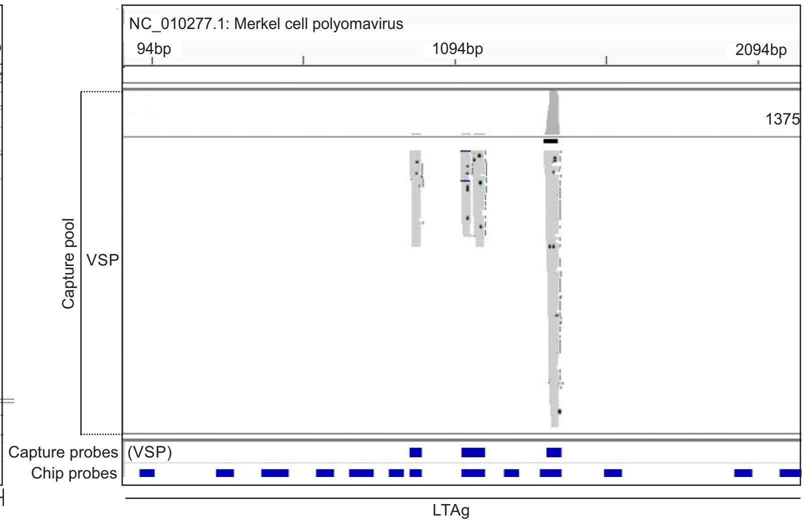

Papillomaviridae

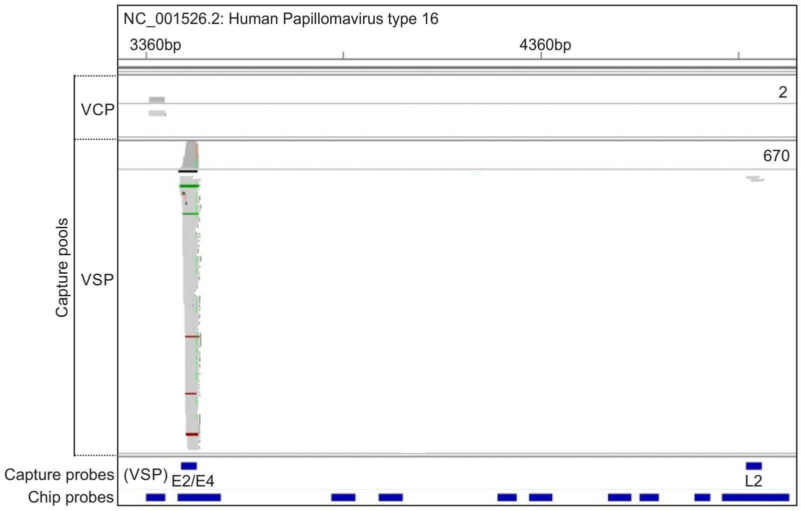

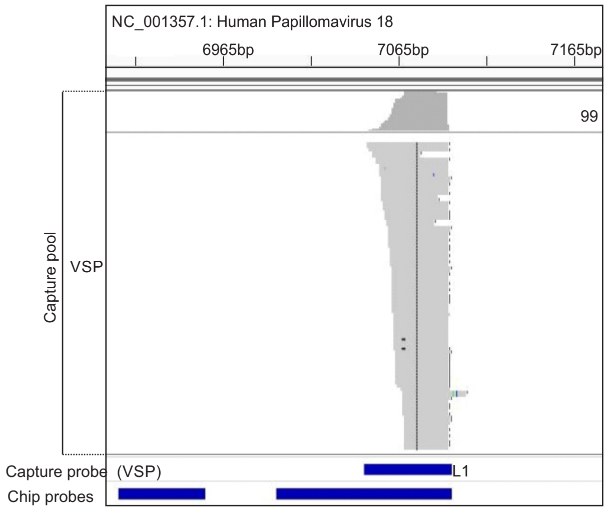

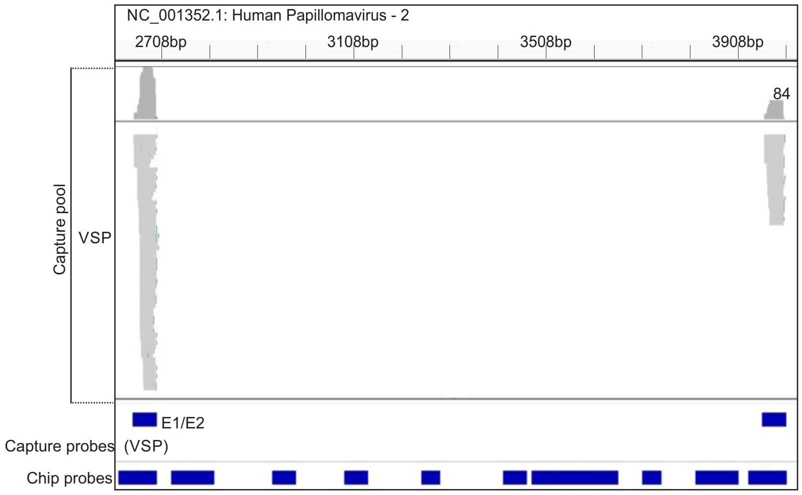

Supplementary Figure S2

Viral Signatures

Plant Viral signatures

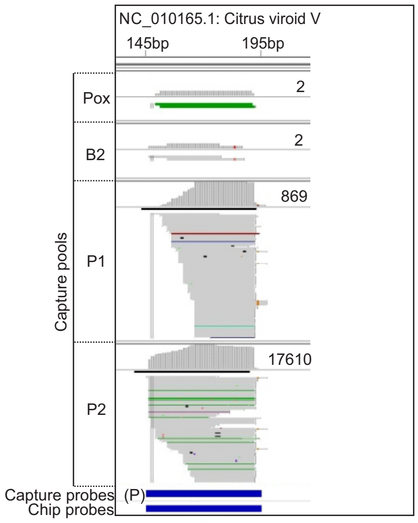

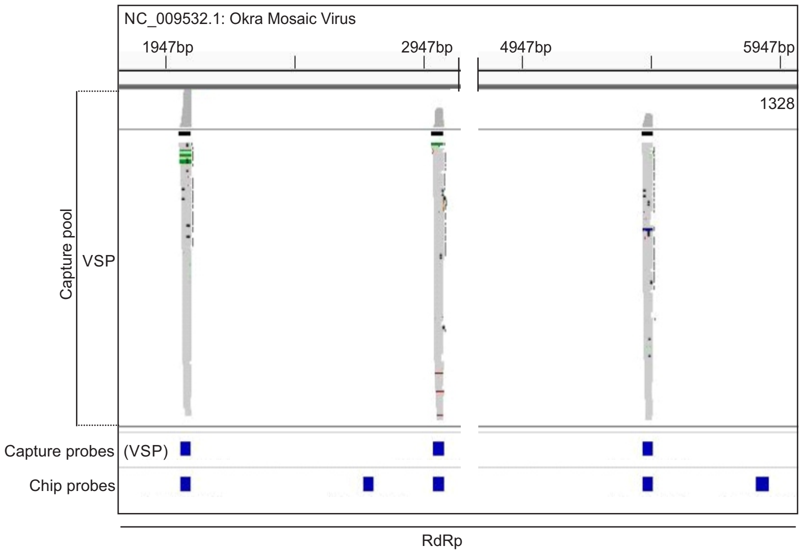

Baculoviridae

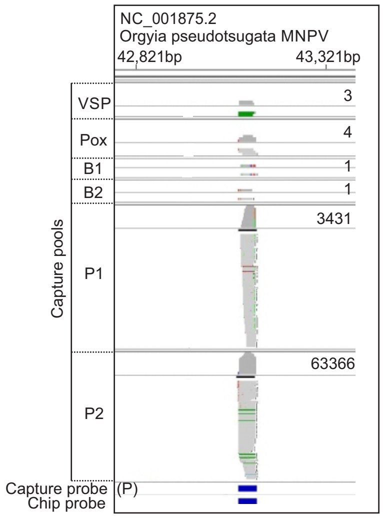

Supplementary Figure S2

Microbial Signatures

Bacterial signatures

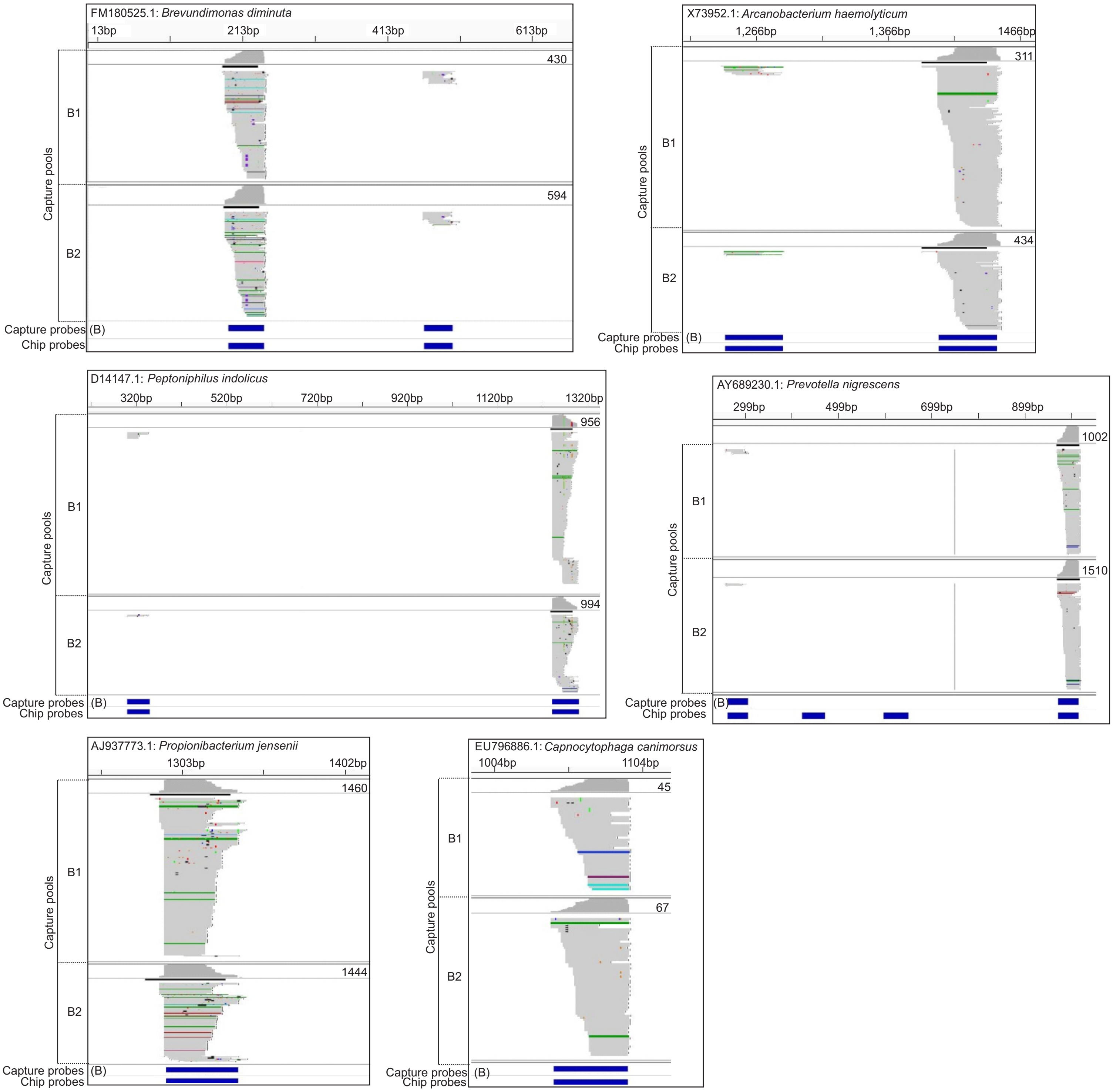

Supplementary Figure S2

Microbial Signatures

Fungal signatures

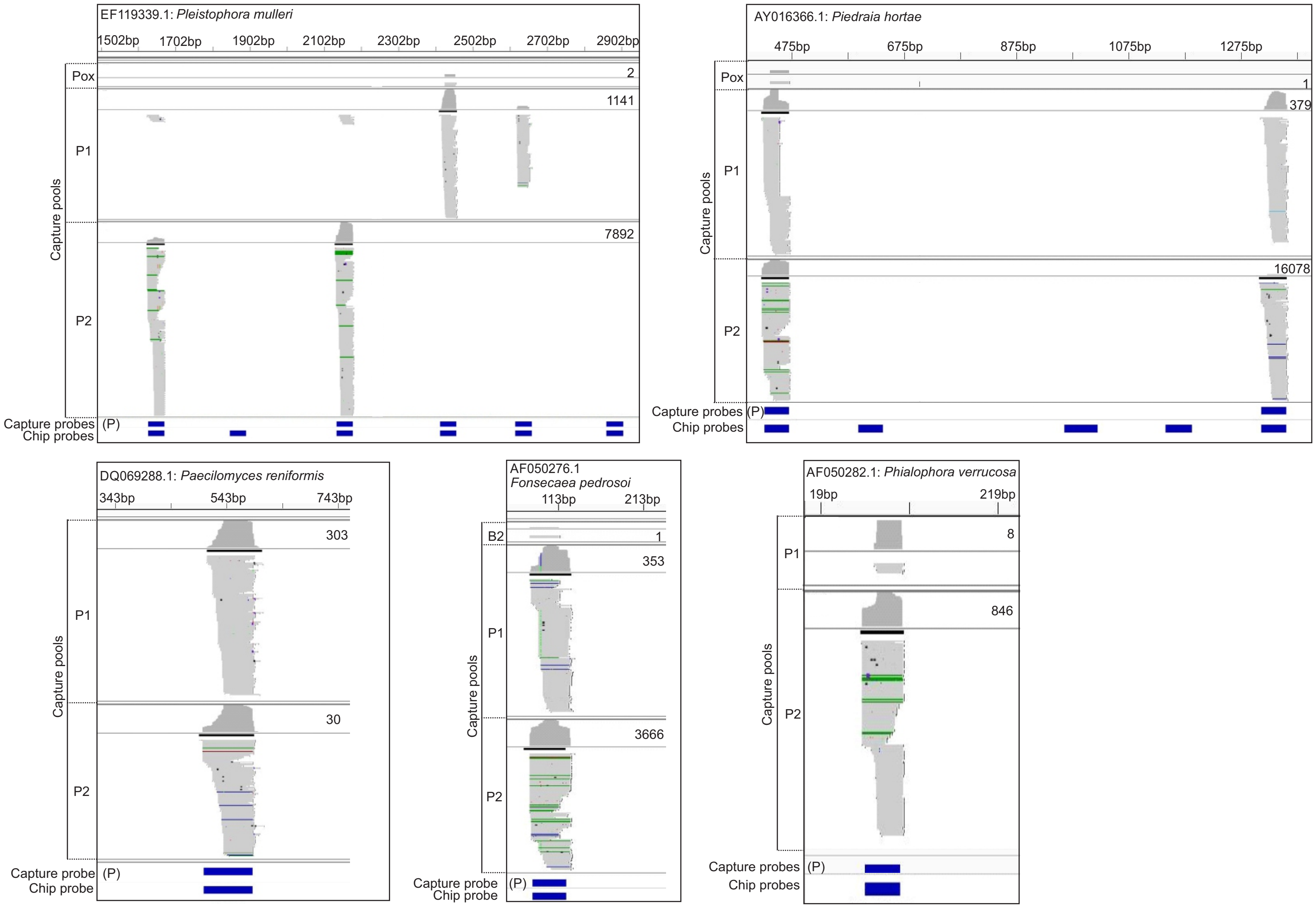

Parasitic signatures

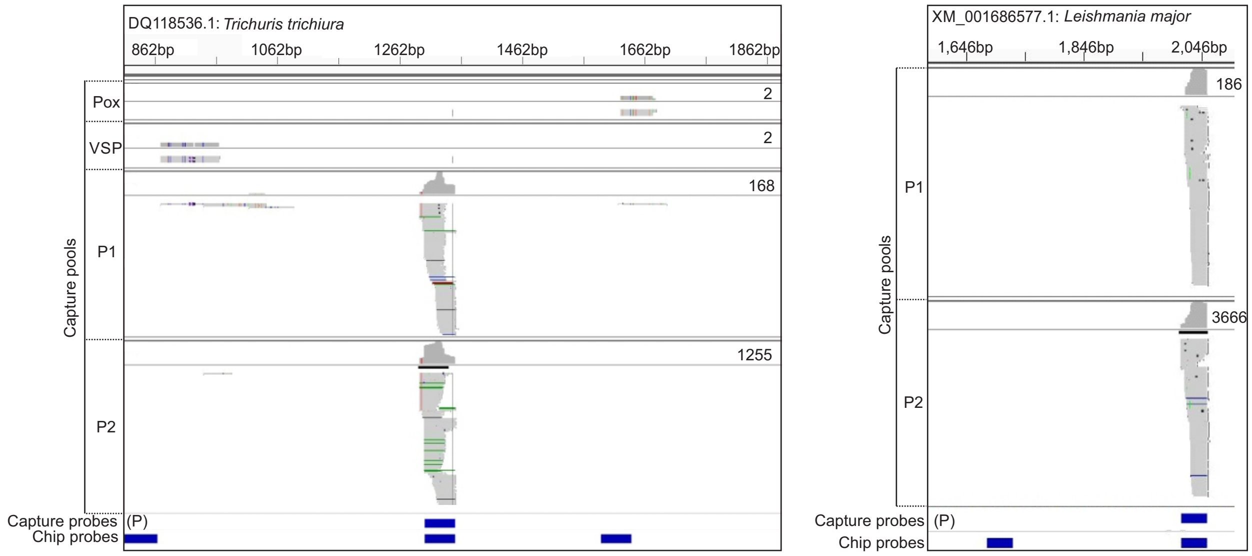

Supplementary Table S1. Percent probes of micro-organisms detected in breast cancer samples versus the controls

Types	Micro-organisms	(p- value)				Average			
		All cases vs. MC	All cases vs. NC	12 pooled cases vs. MC	12 pooled cases vs. NC	All cases	12 pooled cases	MC	NC
Viruses	MMTV	0.00081	0.00049	0.00055	0.00213	31	33	3	0
	Hepatitis C1	0.00154	0.00049	0.0022	0.00213	42	47	3	0
	EBV1	0.00231	0.00049	0.00377	0.00213	31	47	3	0
	BPSV	0.00107	0.00049	0.00451	0.00212	17	15	1	0
	HCMV	0.00162	0.0005	0.00761	0.00213	42	41	5	1
	KSHV	0.00091	0.0005	0.00379	0.00217	39	40	4	1
	PCPV	0.00281	0.00052	0.01926	0.00249	17	14	2	0
	HPV2	0.00091	0.00052	0.00316	0.00213	38	42	2	1
	HTLV-2	0.00073	0.00052	0.00216	0.00212	14	17	1	0
	HPV6B	0.00049	0.00055	0.00214	0.00303	24	22	1	1
	MCPV	0.00061	0.00055	0.00216	0.00212	29	33	2	1
	HTLV1	0.00309	0.00055	0.00316	0.00214	47	60	7	1
	HPV18	0.00101	0.00055	0.00643	0.00308	28	26	2	1
	Hepatitis B	0.00294	0.00058	0.00633	0.00209	41	43	9	2
	SV40	0.00138	0.00061	0.00314	0.00203	35	42	2	0
	HPV16	0.001	0.00069	0.0031	0.00212	24	24	1	12
	HHV1	0.013	0.00112	0.07042	0.00751	37	29	7	0
	Okra Mosaic Virus	0.00331	0.00114	0.00436	0.00199	43	58	4	0
	FSV	0.00061	0.00137	0.00153	0.00234	48	55	0	5
	Hepatitis GB	0.0009	0.00146	0.00755	0.01536	35	36	0	18
	MMLV	0.0025	0.0025	0.01252	0.01252	2	1	0	0
	Viroids	0.00298	0.00298	0.00229	0.00229	50	44	0	0
	Orf Virus	0.00333	0.00333	0.03122	0.03122	12	5	0	0
Bacteria	*Prevotella*	0.00412	0.00099	0.00541	0.00183	42	46	8	0
	Brevundimonas	0.0047	0.00155	0.04501	0.01124	55	36	8	0
	Arcanobacterium	0.00422	0.00181	0.00364	0.00173	53	52	6	0
	Escherichia	0.00234	0.00234	0.03461	0.03461	24	15	0	0
	Sphingobacterium	0.0024	0.0024	0.00562	0.00562	48	53	5	5
	Actinomyces	0.01282	0.00376	0.06882	0.02109	39	29	6	0
	Rothia	0.11245	0.00784	0.0887	0.01193	34	44	17	0
	Mobiluncus	0.30567	0.01263	0.91693	0.11578	38	17	25	0
	Propiniobacter	0.01301	0.01301	0.05107	0.05107	45	29	0	0
	Geobacillus	0.03372	0.03372	0.3325	0.3325	24	4	0	0
	Providencia	0.00419	0.04773	0.07605	0.30672	67	42	0	25
	Peptinophillus	0.06186	0.06186	0.3325	0.3325	35	8	0	0
	Capnocytophaga	0.07289	0.07289	0.3325	0.3325	37	8	0	0
Fungi	*Pleistophora*	0.00154	0.00069	0.00972	0.00292	63	50	9	6
	Paecilomyces	0.0049	0.00173	0.0031	0.00173	52	63	6	0
	Piedra	0.01329	0.00348	0.42648	0.24689	60	35	25	17
	Foncecaea	0.03374	0.01324	0.40045	0.32769	71	53	42	33
	Phialophora	0.13135	0.0147	0.52492	0.32886	50	38	38	25
Parasites	*Trichuris*	0.00159	0.00083	0.00562	0.00328	60	51	18	11
	Babesia	0.01007	0.00333	0.17174	0.05315	40	15	5	0
	Leishmania	0.0041	0.0041	0.01294	0.01294	38	40	0	0
	Toxocara	0.00922	0.00922	0.3325	0.3325	62	8	0	0
	Thelazia	0.05771	0.05771	1	1	40	0	0	0

MC: matched controls; NC: non-matched controls

Supplementary Table S2. Primers used for PCR validation of PathoChip screen.

	Primers	Sequence (5'-3')	Annealing temp and time	Extension temp and time	Amplicon size (bp)
Herpes	FP 1	GAA GAC GCT GAT GAA CCA CG	51^{0}C for 45s	65^{0}C for 20s	96
	RP 2	AAG CAC CTG GTG TAC TTT CAC			
MMTV	FP 3 (Env)	TTA GGG GAG AAG CAG CCA AGG	55^{0}C for 30s	65^{0}C for 30s	184
	SN RP 4 (Env)	AAA GAG TCA AGG GTG AGA GCC			
	RP (Env)	CTT GTA AGA GGA AGT TGG CTG TGG			
MMTV	FP (gag)	CAC AGA TTG GAA CGA TGA TGA CCT G	57^{0}C for 30s	65^{0}C for 30s	70
	SN FP 5 (gag)	ACT CAG AAG GAA ACC CCT GCC TC			
	RP 6 (gag)	ATC TCC TTT TTC CCT GGC CTC TGC			
HPV	FP 7	CTT GAC ATT GTG TGT CCT GCC TG	53^{0}C for30s	65^{0}C for 30s	160
	RP 8	TAA TTC AAA GGT GTC TGC CTC CTG C			
SV40	FP 9	CAG TAG CCT CAT CAT CAC TAG ATG	51^{0}C for 45s	65^{0}C for 20s	94
	RP 10	GGA ACT GAT GAA TGG GAG CAG T			
Parapox	FP 11 (Orf)	ATC TTC ACG GGC GCA GTC G	56^{0}C for 30s	65^{0}C for 30s	286
	RP 12 (Orf)	CTC TTC GAC GAC GAC GGG AAC			
	FP 13 (PcP)	TCGTGATCTCGGTGTCCACCTG	56^{0}C for 30s	65^{0}C for 30s	524
	RP 14 (PcP)	CAT CAA CTA CCT GCT CGA CAG CAC			
MCPV	FP 15	CAG AGA GGA GAC CAC CAA TTC AG	52^{0}C for 45s	65^{0}C for 30s	264
	RP 16	GTG AAG GAG GAG GAT ATG TAT TCC			
Bacteria	FP 17	TTG CAG AGG ACA ATC CGA ACT GAG	52^{0}C for 60s	65^{0}C for 60s	667
	RP 18	AAC TGC CTT TGA TAC TGG CGA TC			
Fungus	FP 19	AGG TCT CCT AGG TGA ATA GCC	48^{0}C for 30s	65^{0}C for 30s	219
	RP 20	CCG TGC TTA CAG TTA TTT CCT C			
Parasite	FP21	GAG GTA GTG ACG AAA AAT AAC GG	48^{0}C for 30s	65^{0}C for 30s	250
	FP22	CCA GAG TCT CGT TCG ATA TCG			

Supplementary Table S4. Number of reads generated in MiSeq.

Libraries	Total reads	Trimmed reads (removing low-quality reads)	Reads mapped to human genome	Reads not mapped to human	Reads mapped to pathogenome	Reads mapped to pathogenome with quality score MapQ>= 20
VCP	1041326	967810	713524	254286	126826	30042
VSP	1186563	1114046	913188	200858	12545	7715
Pox	1203986	1143755	896208	247547	19529	7265
B1	579207	542245	128343	413902	164849	12813
B2	717949	671654	191078	480576	193946	14051
P1	1324969	1239586	986414	253172	21388	10532
P2	689316	646007	208228	437779	141451	120487
Total	6743316	6325103	4036983	2288120	680534	202905

Supplementary Figure Legends.

Supplementary Figure S1. The percent probes of candidate organisms showing undetectable, low (>30 to 300), moderate (300-3000) and high (>3000) hybridization signal (Cy3-Cy5) in breast cancer samples (40 individual and 12 pooled) by PathoChip screening. Matched controls (MC) and non-matched controls (NC) are included to show the significant detection of probes in the breast cancer samples vs the controls. The percentage of specific probes of viral candidates and that of bacterial, fungal and parasitic candidates detected in breast cancer samples with low, medium and high hybridization signal are shown.

Supplementary Figure S2. The miSeq reads aligned to the metagenome of the PathoChip revealing the identity of the targets captured by the selected probes (probe pool VCP, probe pool VSP, probe pool Pox, probe pool B1 and B2, probe pool P1 and P2) during capture sequencing. The genomic location of individual accessions, along with the number of miSeq reads for individual captures are mentioned. The alignment track of IGV displayed the upper coverage track and the lower alignment track. IGV display the paired-end alignments that deviate from expectations by standard color (horizontal colored lines). The mismatched bases are also displayed in color (A as green, C as blue, G as yellow and T as red) on the grey aligned sequence bar that represents the read. The viral signatures and the other microbial signatures captured by the selected probes during capture sequencing are shown.

Legends for Supplementary Tables.

Supplementary Table S1. Percent probes of micro-organisms detected in breast cancer samples versus the controls.

The table shows the statistical significance of percent probes of candidate organisms detected in triple negative breast cancer samples vs. the matched and non-matched control samples. The significance is determined by Wilcoxon tests, and the percent detection of the pathogenic signatures in the cancer tissues were considered significant compared to the control tissues if the p value <0.05.

Supplementary Table S2. Primers used for PCR validation of PathoChip screen.

Supplementary Table S3. Probes used for target capture.

Supplementary Table S4. Number of reads generated in MiSeq.

Supplementary Table S5. Aligned miSeq reads of captured targets. The miSeq reads of candidates in 7 different capture reactions namely bacterial (B1 and B2), parasitic-fungal-viroid (P1 and P2), pox conserved (pox), viral specific (VSP) and viral conserved (VCP) probe. We summarized the reads that map to each organism across the 7 capture sequencing (B1, B2, P1, P2, Pox, VCP and VSP, respectively). Specifically we count the total numbers of reads that aligned to the whole species (*_org), to the capture probe regions (*_probe), and to the out-of-probe regions (*_outprobe). Take the organism DQ118536.1, detected by P1 capture sequencing, for example. There are 168 reads (P1_org) aligned to this organism, of which 160 reads (p1_probe) aligned to the capture probe region and the remaining 8 reads (P1_outprobe) aligned to out-of-capture-probe regions. For each organism, the score column gives the number of capture sequencing under which reads are mapped to both the capture probe regions and the out-

of-probe regions. For example, the score of organism DQ118536.1 is 2 because we find reads mapped to both the probed regions and out-of-probe regions by P1 and P2 capture sequencing. We also summed up the total number of reads mapping to the capture probe regions in all the 7 capture sequencing conditions in the Probe_score column. We list those candidate organisms with reads that mapped to the capture probe regions (Probe_score>0) and rank them by the score column.
